# Comparative spatial lipidomics analysis reveals cellular lipid remodelling in different developmental zones of barley roots in response to salinity

**DOI:** 10.1111/pce.13653

**Published:** 2019-11-29

**Authors:** Lenin D. Sarabia, Berin A. Boughton, Thusitha Rupasinghe, Damien L. Callahan, Camilla B. Hill, Ute Roessner

**Affiliations:** ^1^ School of BioSciences and Metabolomics Australia University of Melbourne Parkville VIC 3010 Australia; ^2^ School of Life and Environmental Sciences, Centre for Chemistry and Biotechnology, (Burwood Campus) Deakin University, Geelong, Australia 221 Burwood Highway Burwood VIC 3125 Australia; ^3^ School of Veterinary and Life Sciences Murdoch University Murdoch WA 6150 Australia

**Keywords:** barley, glycerophosphocholine, lipids, MALDI, mass spectrometry imaging, metabolome, salinity, spatial metabolomics

## Abstract

Salinity‐induced metabolic, ionic, and transcript modifications in plants have routinely been studied using whole plant tissues, which do not provide information on spatial tissue responses. The aim of this study was to assess the changes in the lipid profiles in a spatial manner and to quantify the changes in the elemental composition in roots of seedlings of four barley cultivars before and after a short‐term salt stress. We used a combination of liquid chromatography–tandem mass spectrometry, inductively coupled plasma mass spectrometry, matrix‐assisted laser desorption/ionization mass spectrometry imaging, and reverse transcription – quantitative real time polymerase chain reaction platforms to examine the molecular signatures of lipids, ions, and transcripts in three anatomically different seminal root tissues before and after salt stress. We found significant changes to the levels of major lipid classes including a decrease in the levels of lysoglycerophospholipids, ceramides, and hexosylceramides and an increase in the levels of glycerophospholipids, hydroxylated ceramides, and hexosylceramides. Our results revealed that modifications to lipid and transcript profiles in plant roots in response to a short‐term salt stress may involve recycling of major lipid species, such as phosphatidylcholine, via resynthesis from glycerophosphocholine.

## Abstract 2

Accumulation of glycerophosphocholine may be a component in the salt stress response of seminal roots of barley seedlings by acting as an osmolyte and a substrate for phosphatidylcholine resynthesis following a short‐term salt stress.

## 1 INTRODUCTION

Barley (*Hordeum vulgare*) is the most ecologically diverse grain worldwide; it is used as a forage grain, staple food, and malt for brewing (Adem, Roy, Zhou, Bowman, & Shabala, 2014; Meng et al., 2016). Among cereals, barley is the most tolerant to salt stress, with different varieties and cultivars differing in their tolerance (Adem et al., 2014; Chen et al., 2007; Dai et al., 2012). Understanding the physiological and metabolic mechanisms that confer salt tolerance of barley is of agronomic and economic interest. The discovery of tissue tolerance traits could be used to select more salt‐tolerant varieties that maintain high yield under salt stress, and any positive traits could be transferred to other commercially important crops such as maize, wheat, and rice.

Lipids are the major structural components of plasma and endomembranes and have recognizable structural roles in response to abiotic stresses such as salinity (Barrero‐Sicilia, Silvestre, Haslam, & Michaelson, 2017). Plant lipids encompass a variety of molecular species with great structural diversity ranging from simple free fatty‐acid molecules to more complex sphingolipids (Barrero‐Sicilia et al., 2017). Plant lipids have unique and varying physical properties conferring plants a wide range of biological functions, including energy storage, surface protection, structural, and signalling roles. Cell membranes are key elements that provide protection and aid in homeostasis maintenance within plant cells. The main components of plant membranes are glycerolipids, glycerophospholipids, and sterol lipids (Hu, Yu, Chen, & Li, 2018).

Although many studies exist on the effects of salinity on crop growth, few have investigated root metabolic profiles (Annunziata et al., 2017; Cao, Lutz, Hill, Callahan, & Roessner, 2016; Gavaghan et al., 2011; Geilfus et al., 2015; Guo et al., 2017; Jiao et al., 2018; Richter, Erban, Kopka, & Zörb, 2015), and an even smaller number have conducted lipidomics analyses of salt‐stressed root tissues (Natera, Hill, Rupasinghe, & Roessner, 2016; Yu et al., 2018). Further, most of these comparative studies are based on bulked tissue extractions that often mask tissue‐specific metabolic differences. Plant biochemistry and physiology are spatially segregated at the subcellular and multicellular level (Sumner, Lloyd W, 2010), thus, there is an increasing interest in investigating plant tissue‐specific responses to changing environments in a spatially resolved manner through tissue specific sectioning or by using mass spectrometry imaging (MSI)‐based techniques (Sarabia *et al*., 2018b). MSI‐based metabolomics allow assessment of the functional roles of plant metabolites by measuring their distribution *in situ* and exploring the localized tissue and cell‐specific response to abiotic stresses (Boughton, Thinagaran, Sarabia, Bacic, & Roessner, 2016).

Spatially resolved metabolomics (Shelden, Dias, Jayasinghe, Bacic, & Roessner, 2016) and transcriptomics (Hill et al., 2016) were recently applied to analyse the molecular effects of a short‐term salt stress in seminal roots of barley seedlings. In both studies, root tissue was dissected into different developmentally distinct zones: root cap and zone of cell division (S1), zone of cell elongation (S2), and zone of cell maturation (S3), revealing spatial metabolite or gene expression gradients along the different developmental zones of the barley root. In our previous work (Sarabia *et al*., 2018a), we developed a matrix‐assisted laser desorption/ionization MSI (MALDI‐MSI) method to analyse the spatial distribution of lipids and metabolites in longitudinal sections of roots of the barley cultivar Hindmarsh and applied this to investigate spatially resolved molecular changes in response to a short‐term salt stress. Building on this study, we have applied the MALDI‐MSI method in combination with liquid chromatography–electrosprayionization–tandem mass spectrometry and inductively coupled plasma mass spectrometry (ICP‐MS) analyses to comparatively analyse the lipidomes and elemental profiles of four different barley cultivars, which differ in their tolerance to salinity (Shelden, Roessner, Sharp, Tester, & Bacic, 2013; Widodo, Newbigin, Tester, Bacic, & Roessner, 2009). To investigate the spatially localized molecular signatures of salinity stress, we analysed changes in the composition, distribution, and saturation levels of several lipid species in dissected root tissues in response to a short‐term high salt (150 mM of NaCl) stress using liquid chromatography–mass spectrometry (LC‐MS) as well as MALDI‐MSI‐based lipidomics. We identified major metabolic changes, such as increases in the levels of phosphatidylcholine (PC) and glycerophosphocholine (GPC), which were hypothesized to be an adaptive response of barley to saline conditions. To provide evidence on the dynamics of PC metabolism in the maintenance of membrane integrity and fluidity in response to short‐term salt stress, we employed Reverse Transcription quantitative real time polymerase chain reaction (RT‐qPCR) analysis to investigate the changes in the relative gene expression of five genes involved in PC metabolism pathways and their intermediates. Our study contributes new insights on salinity stress responses along different developmental barley root zones and suggests sites of lipid regulation that can be attributed to short‐term salinity stress.

## 2 MATERIALS AND METHODS

### 2.1 Plant material and experimental conditions

Seeds of domestic barley (*H. vulgare* L.) malting cultivars Clipper and Gairdner, food grade cultivar Hindmarsh, and the feed cultivar Mundah were grown in petri plates containing modified Hoagland solution in a climate‐controlled growth chamber under a cycle of 17°C without fluorescent light for 48 h as previously described (Hill et al., 2016; Shelden et al., 2016). Salt stress treatment was achieved by adding NaCl to a final concentration of 150 mM of NaCl to the Hoagland solution.

For LC‐MS/MS and RT‐qPCR, seminal roots were dissected per root zones described in Table S1, snap‐frozen, and then stored at −80°C. For MALDI‐MSI, Barley seminal root were excised, embedded in a SCEM (super cryo‐embedding medium) matrix, and frozen using a slurry mixture of dry ice:isopropanol as previously described (Sarabia *et al*., 2018a).

#### 2.1.1 Lipid extraction and LC‐MS/MS analysis

Lipid extraction and analysis was carried out for four biological replicates per cultivar and treatment, as previously described (Shiva et al., 2018). Briefly, lipids were extracted from ~25 mg of homogenized root tissue by suspending the ground tissue in 400 μL of cold isopropanol containing 0.01% butylated hydroxytoluene spiked with 50‐μM deuterated cholesterol. The samples were homogenized using a cryo‐mill (Bertin Technologies; Montigny‐le‐Bretonneux, France) for 3 cycles at 6,100 rpm with 45 s on and 45 s rest between cycles at −10°C and was then incubated at 75°C whilst shaking at 1,400 rpm for 15 min. After 1,200 μL of chloroform:methanol:water (30:41.5:3.5, v/v/v) were added to a final solvent concentration of chloroform:isopropanol:methanol:water (30:25:41.5:3.5, v/v/v/v), the samples were shaken at 300 rpm at 25°C for 24 h and centrifuged at 13,000 rpm for 15 min at room temperature. The solvent was collected in a new 2.0‐ml tube and dried in a vacuum evaporator. The pellet was resuspended in 200 μL of methanol:butanol (1:1, v/v) containing 20 mM of ammonium acetate of which 10 μL were subjected to LC‐MS/MS analysis (Yu et al., 2018). As many lipid classes can differently ionize in either electrospray ionization+ or electrospray ionization− using LC‐MS, we analysed lipid extracts obtained from dissected root sections consecutively in both positive and negative ionization modes.

### 2.2 Lipid identification and statistical analysis

The LC‐MS/MS data were processed using MultiQuant™ 3.0.2 Software (SCIEX; Framingham, MA, USA), identified using an in‐house‐generated lipid database for barley (Yu et al., 2018). The data were normalized to the sample fresh weight. We compared the signal intensities of observed ions, expressed as peak area for each of the identified lipid compounds. Statistical analysis of the normalized lipid species was carried out using MetaboAnalyst (Chong et al., 2018). For multiple group analysis, univariate anova and Tukey's honestly significant different test were performed. For pairwise comparative analysis, Student's *t*‐tests were conducted on each individual lipid species/compounds to determine significant differences between two groups. For all analyses, adjusted *p*‐values using Benjamini‐Hochberg false discovery rate correction were considered (Benjamini, Krieger, & Yekutieli, 2006).

### 2.3 *In situ* MALDI‐MSI metabolite analysis

Spatially localized metabolites were analysed from longitudinal sections of seminal roots of four barley cultivar under control and salt treatment. Metabolites were analysed by MALDI Fourier transform ion cyclotron resonance ICR‐MSI (MALDI‐FT‐ICR‐MSI) in both positive and negative ionization mode to enable profiling of several lipid classes and metabolites using a modified method (Data S1) previously described (Sarabia *et al*., 2018a).

#### 2.3.1 Metabolite identification and statistical analysis

The MALDI‐MSI data were processed using SCiLS Lab 2017a (SCiLS, Bremen, Germany). Mass spectra were normalized to the root mean square. Receiver operating characteristic (ROC) analysis was performed using previously identified *m*/*z* intervals (Sarabia *et al*., 2018a). In ROC, control and salt‐treated root sections were further divided into: S1, S2, and S3 (Table S1) to create a region of interest in the imported image in SCiLS Lab. The *m*/*z* values with an area under the curve value >0.7 and <0.3 were considered discriminative in control and salt‐treated roots, respectively. Discriminative *m*/*z* values were searched against the LIPID MAPS (Fahy, Sud, Cotter, & Subramaniam, 2007) and METLIN (Smith et al., 2005) databases to provide tentative peak assignments with a Δ ppm < 0.005 mass error and were visualized as single ion images.

### 2.4 RNA extraction and relative gene expression analysis

A homogenized plant material of ten replicate samples per cultivar and treatment was pooled from 20 dissected root sections separately for Clipper and Hindmarsh cultivars only. Total RNA was isolated using the RNeasy Mini Plant Kit combined with the RNase‐Free DNase Set (QIAGEN; Doncaster, Australia) as per the manufacturer's instructions. RNA quality and integrity were verified using a NanoDrop ND‐2000UV‐Vis Spectrophotometer (Thermo Fisher Scientific; Scoresby, Australia). cDNA was transcribed from 1‐μg total RNA using Superscript™ III Reverse Transcriptase (Thermo Fisher Scientific; Scoresby, Australia) as per the manufacturer's instructions.

Primers for five candidate genes involved in the glycerophospholipid metabolism Kyoto Encyclopedia of Genes and Genomes pathway (https://www.genome.jp/kegg/pathway/map/map00564.html) were generated the Primer3 online tool (http://bioinfo.ut.ee/primer3/) and checked with blast searches against BARLEX (https://webblast.ipk-gatersleben.de/barley_ibsc/) to exclude multiple binding sites (Table S6). RT‐qPCR analysis was performed on a CFX384™ Touch Real Time PCR Detection System (Bio‐Rad; Gladesville, Australia) using 384‐well plates. Reactions of 10 μL of final volume contained 5 μL of 2× SensiFAST™ SYBR® No‐ROX Mater Mix (Bioline; Boston, MA, USA), 2.2 μL of nuclease‐free water, 0.4 μL of each 10 μM of forward and reverse primers, and 2 μL of cDNA. The reaction conditions were as follows: initial denaturation at 95°C for 2 min; followed by 40 cycles of 5 s at 95°C, 10 s at 60°C, and 20 s at 72°C, where the fluorescence signal was measured. Standardization was carried out based on the expression of glyceraldehyde 3‐phosphate dehydrogenase and *S*‐adenosyl‐l‐methionine‐dependent methyltransferase superfamily protein genes in each sample. The relative abundance of transcripts was calculated by using the 2^−ΔΔCt^ method (Livak & Schmittgen, 2001). Negative controls without cDNA were used in all PCR reactions. The primers used are listed in Table S7.

### 2.5 Elemental analysis

Elemental extraction and analysis were performed for three pooled biological replicates per cultivar and treatment, as previously described (Callahan, Hare, Bishop, Doble, & Roessner, 2016). Briefly, micronutrients were extracted from ~10 mg of homogenized dry tissue by digestion with 600 μL of aqua regia for 90 min at 80°C. After digestion, the samples were diluted to 10 ml with deionized water and centrifuged at 5,000 rpm for 5 min prior to analysis by ICP‐MS (NexION 350X, PerkinElmer; Waltham, MA, USA), and Na, K, Mg, Fe, Ca, Mn, Cu, and Zn were measured (Callahan et al., 2016). All analyses were performed using Syngistix™ (PelkinElmer; Waltham, MA, USA) software.

## 3 RESULTS

### 3.1 Seminal root development under short‐term salinity

#### 3.1.1 Whole seminal root

Root growth of the four barley cultivars was significantly inhibited by a short‐term salt stress; however, significant differences were found in the levels of root length inhibition across the four barley cultivars (Figure S1). Root growth was more significantly impacted by salinity in Clipper and Mundah that had a −2.74‐ and −2.42‐fold decrease in root length, respectively. In contrast, Hindmarsh and Gairdner showed a lower inhibition in root length with a −1.83‐ and −1.45‐fold decrease, respectively.

#### 3.1.2 Reduction in length of developmental root zones

Similarly, the root cap and zone of cell division showed a reduction in length of −1.25‐fold, −1.17‐fold, and −1.14‐fold for Mundah, Clipper, and Gairdner, respectively. Whereas all four cultivars showed a reduction in length of the zone of cell elongation of −2.75‐fold, −1.39‐fold, −1.25‐fold, and −1.11‐fold decrease for Clipper, Mundah, Hindmarsh, and Gairdner, respectively (Table S1).

### 3.2 Lipid profiles of developmental root zones across four barley cultivars.

#### 3.2.1 Developmental differences in the lipid profile in seminal roots of barley

##### 3.2.1.1 Differences in the lipid profile between zone of cell division and zone of cell elongation

There were significant differences between the levels of the analysed lipid species between root zones. Developmentally regulated lipid species that were common between the root cap and zone of cell division (S1) and the zone of cell elongation (S2) in all four barley cultivars include 61 lipid species. These lipids include hydroxylated hexosylceramides, acylated sterol glycosides (ASGs), diacylglycerols (DAGs), hydroxylated ceramides (Cer‐OHs), cardiolipins (CLs), and lysophosphatidylcholine (LPC) representing 19.7%, 14.8%, 11.5%, 9.8%, 8.2%, and 4.9% of the developmentally regulated lipids that showed a more than 2‐fold higher content in S1 compared with S2 in both control and salt‐treated roots, respectively (Data S2).

Further, lipid species that were different between S1 and S2 of control roots in all four barley cultivars include two ASG, one ceramide (Cer), two DAG, two digalactosylmonoacylglycerol (DGMG), two LPC, two lysophosphatidylethanolamine (LPE), two lysophosphatidylglycerol (LPG), and one lysophosphatidylserine (LPS). On the other hand, lipid species that were different between S1 and S2 of salt‐treated roots in all four barley cultivars also include one ASG, five Cer, three CL, five DAG, two hexosylceramides (HexCer), one phosphatidylethanolamine (PE), three phosphatidylglycerol (PG), and one phosphatidylinositol (PI; Data S2).

##### 3.2.1.2 Differences in the lipid profile between zone of cell division and zone of cell maturation

There were 121 common developmentally regulated lipid species between S1 and the zone of cell maturation (S3) in all four barley cultivars. These lipid species primarily include DAG, hydroxylated hexosylceramides, ASG, CL, Cer‐OH, Cer, and PG representing 19.0%, 19%, 9.9%, 8.3%, 7.4%, 5.8% and 5.8% of the developmentally regulated lipids that showed a more than 2‐fold higher content in S1 compared with S3 in both control and salt‐treated roots, respectively (Data S2).

Lipid species that were different between S1 and S3 of control roots in all four barley cultivars include two ASG, one DAG, one DGMG, one HexCer, three LPC, two LPE, one LPG, one LPS, four PG, two PI and one sulfoquinovosylmonoacylglycerol (SQMG). Whereas lipid species that were different between S1 and S3 of salt‐treated roots in all four barley cultivars include one Cer, three DAG, one digalactosyldiacylglycerol (DGDG), one HexCer, six PC, four PE, three PG, four PI, and three sulfoquinovosyldiacylglycerol (SQDG; Data S2).

##### 3.2.1.3 Differences in the lipid profile between zone of cell elongation and zone of cell maturation

There were 10 common developmentally regulated lipid species between S2 and S3 in all four barley cultivars. These lipid species include Cer(t18:0_26:0), Cer(t18:0_26:1), Cer(t18:1_24:0), Cer(t18:1_24:0‐OH), Cer(t18:1_24:1‐OH), Cer(t18:1_26:0), DAG(18:0_18:3), DAG(18:3_24:1), HexCer(d18:2_14:0), and HexCer(d18:2_14:0‐OH) that showed a more than 2‐fold higher content in S2 compared with S3 in both control and salt‐treated roots, respectively (Data S2).

Moreover, lipid species that were different between S2 and S3 of control roots in all four barley cultivars include two Cer, four DAG, and three HexCer. Whereas lipid species that were different between S2 and S3 of salt‐treated roots in all four barley cultivars include two Cer, one DAG, one DGDG, two PC, one PE, three PG, three PI, and two SQDG (Data S2).

#### 3.2.2 Salinity effects on lipid profiles show distinct signatures between different barley cultivars

To elucidate the effect of salinity on lipid profiles along the roots, we analysed lipid extracts obtained from dissected sections of three different developmental root zones of four barley cultivars, namely malting varieties Clipper and Gairdner, food cultivar Hindmarsh, and feed cultivar Mundah.

##### 3.2.2.1 Root cap and zone of cell division

**Figure 1:**
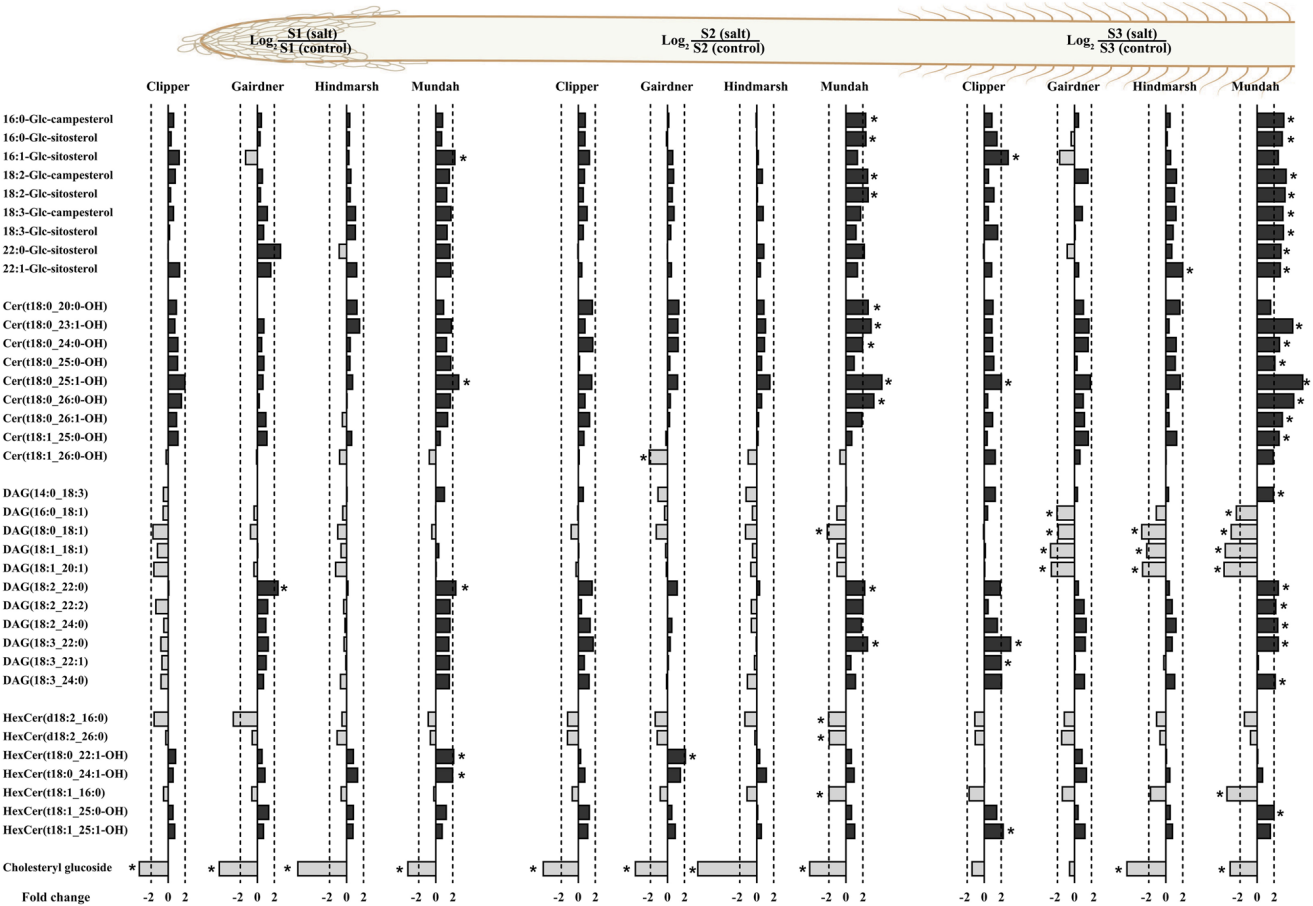
Log2 ratios of representative sterol glucosides, diacylglycerols, ceramides, and hexosylceramides content in roots of barley cultivars Clipper, Gairdner, Hindmarsh, and Mundah that are salt grown (150 mM of NaCl) compared with control grown. Values that are significantly higher (*P* < .05, false discovery rate) are indicated with one asterisk. A threshold of ±2‐fold change is indicated by a dashed line. Image displays lipid contents of three developmental root zones: S1: root cap and zone of cell division, S2: zone of cell elongation, and S3: zone of cell maturation [Colour figure can be viewed at http://wileyonlinelibrary.com]

**Figure 2:**
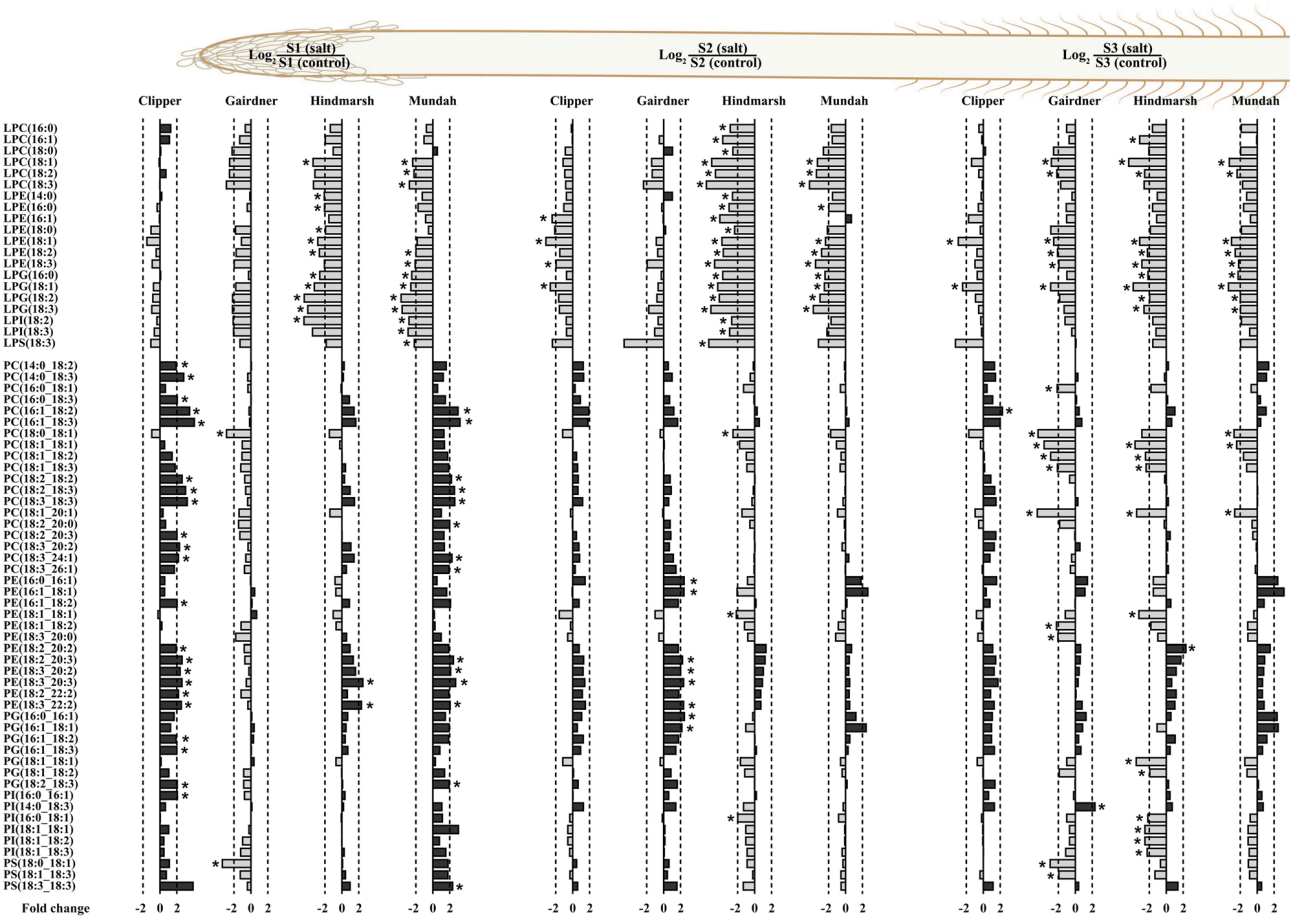
Log2 ratios of representative lysoglycerophospholipids and glycerophospholipids in roots of barley cultivars Clipper, Gairdner, Hindmarsh, and Mundah that are salt grown (150 mM NaCl) compared with control grown. Values that are significantly higher (*P* < .05, false discovery rate) are indicated with one asterisk. A threshold of ±2‐fold change is indicated by a dashed line. Image displays lipid contents of three developmental root zones: S1: root cap and zone of cell division, S2: zone of cell elongation and S3: zone of cell maturation [Colour figure can be viewed at http://wileyonlinelibrary.com]

The targeted lipid profile of the root cap and zone of cell division zone (S1) revealed several changes across four barley cultivars. Mundah and Clipper showed the larger number of lipid species that were significantly increased by more than a 2‐fold change after salt stress. Among the common lipid species found significantly increased in both Mundah and Clipper were PC(16:1_18:2), PC(16:1_18:3), PC(18:2_18:2), PC(18:2_18:3), PC(18:3_18:3), PE(16:1_18:2), PE(18:2_20:3), PE(18:3_20:2), PE(18:3_20:3), PE(18:3_22:2), PG(18:2_18:3), SQDG(16:1_18:2), SQDG(18:2_18:2), SQDG(18:2_18:3), and SQDG(18:3_18:3; Data S3, Figures 1 and 2).

Cholesteryl glucoside was the only lipid that exhibited a significant decrease of more than 2‐fold after salt stress in all the four barley cultivars. Furthermore, Mundah, Gairdner, and Hindmarsh showed the larger number of lipids that were significantly decreased by more than a 2‐fold change after salt stress. The decreased lipids common to both Mundah and Hindmarsh include DGMG(18:1), DGMG(18:3), LPC(18:1), LPC(18:2), LPC(18:3), LPE(18:2), LPE(18:3), LPG(16:0), LPG(18:1), LPG(18:2), LPG(18:3), and lysophosphatidylinositol (LPI) LPI(18:2). By contrast, common lipids between Gairdner and Hindmarsh exhibited the reduction of LPC(20:1) and LPC(20:2) (Data S3, Figures 1 and 2).

##### 3.2.2.2 Zone of cell elongation

The lipid content of the zone of cell elongation (S2) showed a significant increase of more than 2‐fold in the relative amount of 15, 14, 4, and 2 lipid species in Gairdner, Mundah, Clipper, and Hindmarsh, respectively. SQDG(16:1_18:1) and monogalactosyldiacylglycerol (MGDG) MGDG(16:1_16:1) were found significantly increased in both Clipper and Gairdner after salt stress (Data S3, Figures 1 and 2).

Cholesteryl glucoside showed a significant decrease of more than 2‐fold in all four barley cultivars after salt stress. Hindmarsh and Mundah showed the larger number of significantly decreased lipids by more than 2‐fold after salt stress. The common lipids showing a significant reduction between Mundah and Hindmarsh include DGMG(18:1), LPC(18:0), LPC(18:1), LPC(18:2), LPC(18:3), LPE(18:2), LPG(16:0), LPG(18:2), LPG(18:3), LPI(18:3), LPS(18:2), LPS(18:3), monogalactosylmonoacylglycerol MGMG(18:2), SQMG(18:0), SQMG(18:2), and SQMG(18:3). Common lipids between Mundah, Clipper, and Hindmarsh include LPE(18:1), LPE(18:3), and LPG(18:1). Furthermore, MGDG(18:1_18:1) was the only lipid common between Mundah and Clipper (Data S3, Figures 1 and 2).

##### 3.2.2.3 Zone of cell maturation

The lipid content of the zone of cell maturation (S3) showed a significant increase of more than 2‐fold in the relative amount of 25, 8, 2, and 1 lipid species in Mundah, Clipper, Hindmarsh, and Gairdner, respectively. Common lipids found between Mundah and Clipper with an increase of more than 2‐fold include ASG 16:1‐Glc‐sitosterol, Cer(t18:0_25:1‐OH), DAG(18:3_22:0), and DAG(18:3_24:0; Data S3, Figures 1 and 2).

S3 showed a significant decrease in the levels of DGMG(18:1), LPE(18:1), LPG(18:1), and MGDG(18:1_18:1) in all four barley cultivars after a short‐term salt stress. In contrast, Mundah, Gairdner, and Hindmarsh showed a common significant decrease in the levels of DAG(18:0_18:1), DAG(18:1_18:1), DAG(18:1_20:1), LPC(18:1), LPC(18:2), LPE(18:2), LPE(18:3), PC(18:0_18:1), PC(18:1_18:1), PC(18:1_20:1), SQDG(18:1_18:1), and SQMG(18:2) after salt stress. Mundah and Hindmarsh showed the decrease in levels of LPC(20:1), LPG(16:0), LPG(18:2), LPG(18:3), and SG. cholesteryl glucoside. Further, Mundah and Gairdner had a decrease in the levels of DAG(16:0_18:1) and MGDG(18:0_18:2), whereas Gairdner and Hindmarsh had a decrease in the levels of LPC(22:1), PC(18:1_18:2), PC(18:1_18:3), SQDG(18:1_18:2) and SQDG(18:1_18:3; Data S3, Figures 1 and 2).

### 3.3 MALDI‐MSI analysis of barley roots

We used MALDI‐MSI to determine changes in the spatial metabolite distribution in seminal roots of four barley cultivars following exposure to 150 mM of NaCl for 48 h. Comparisons of the annotated lipid species between the imaged sections of whole roots grown under control and salt‐treated conditions revealed different numbers of discriminative lipids in the four barley cultivars. On the other hand, PI‐Cer[d18:0/16:0(2OH)] was the only lipid that showed a higher relative intensity in control roots of Clipper and Gairdner. By comparison, common lipids with a higher relative intensity in the salt‐treated roots include (+)‐bornyl‐diphosphate, 1,13‐dihydroxy‐herbertene, and (9*R*,13*R*)‐1a,1b‐dihomo‐jasmonic acid for both Clipper and Gairdner, and 7*S*,8*S*‐epoxy‐17*R*‐hydroxy docosahexaenoic acid (HDHA) for Gairdner and Hindmarsh (Table S2).

Further, the anatomical differences between control and salt‐treated roots listed in Table S1 were considered for comparison between control and salt‐treated roots to ensure discriminative results provide a close estimate of the differences in the relative intensity of the analysed lipid species.

#### 3.3.1 Discriminative lipid species found in the root cap and zone of cell division

Common lipid species with a higher relative intensity in the control root cap and zone of cell division include Mayolene‐19, LPA(18:2), LPE(18:3), LPE(18:2), LPG(18:3), LPI(18:3), and LPI(18:2) species in both Gairdner and Mundah; PI‐Cer(d20:1/16:0) and 14R,21R‐diHDHA in both Clipper and Mundah; Carthamidin and Oxyresveratrol in both Gairdner and Hindmarsh; and Lipoic acid Palmitic acid in Clipper, Hindmarsh, and Mundah. Further, Clipper and Hindmarsh shared LPA(16:0), PG(32:0), PG(16:0), LPE(16:0), OH‐Chlorobactene glucoside, and phosphatidic acid (PA) PA(34:1)**.** Regarding common lipids with a higher relative intensity in the salt‐treated root cap and zone of cell division, Clipper and Mundah had PC(36:5), PC(36:6), PC(36:7), PC(38:8), and PC(38:9); Clipper and Gairdner had (+)‐bornyl‐diphosphate, 1,13‐dihydroxy‐herbertene and prebarbigerone; and Clipper and Hindmarsh had LPC(18:2; Table S3.

#### 3.3.2 Discriminative lipid species found in the zone of cell elongation

Common lipid species with a higher relative intensity in control zones of cell elongation include sorbitan palmitate between Clipper and Hindmarsh, 14*R*,21*R*‐diHDHA between Clipper and Gairdner, and PI‐Cer(d18:1_14:0) between Clipper and Mundah. Additionally, there were only three common lipid species with a higher relative intensity in salt‐treated root sections with PC(34:3) and PC(38:9) between Clipper and Hindmarsh and (+)‐bornyl‐diphosphate between Clipper and Gairdner (Table S4).

#### 3.3.3 Discriminative lipid species found in the zone of cell maturation

Common lipid species with a higher relative intensity in the control zone of cell maturation of Gairdner and Hindmarsh include docosatrienoate, 10‐octadecenoic acid, 10,13‐octadecadienoic acid, alpha‐linolenic acid, PA(36:3), PE(34:3), PE(34:2), PE(36:5), PG(34:2), PG(34:3), PI(34:2), and PI(34:1). For Clipper and Mundah common lipid species include PI‐Cer(d18:1/14:0). Conversely, the number of common lipid species with a higher relative intensity in the salt‐treated zone of cell maturation included (3'‐sulfo)Galbeta‐Cer(d18:1/ 16:0(2OH)) between Hindmarsh and Mundah, (+)‐bornyl‐diphosphate between Clipper and Gairdner, 2'‐hydroxymatteucinol, and 7*S*,8*S*‐epoxy‐17*R*‐HDHA between Gairdner and Hindmarsh (Table S5).

### 3.4 Relative gene expression analysis

Relative gene expression analysis of five genes of interest (GOI): HORVU2Hr1G122470, HORVU3Hr1G023960, HORVU5Hr1G084740, HORVU3Hr1G079900, and HORVU4Hr1G088470 revealed a root‐tissue specific pattern of a decrease in the levels of expression in both Clipper and Hindmarsh. The full description of the five GOI is presented in Table S6 and S7.

Hindmarsh and Clipper cultivars were chosen among the reported four barley cultivars for relative gene expression analysis due to their tolerance to salinity and importance (current and historic) for barley production in Australia (Kamboj, Ziemann, & Bhave, 2014). Further, Clipper is a malting cultivar and Hindmarsh is a food cultivar that were chosen in order to maximize genetic diversity to assess their response to a short‐term salt stress.

#### 3.4.1 Changes in the relative gene expression in sections of seminal roots of Clipper grown under control and salt‐treated conditions.

**Figure 3:**
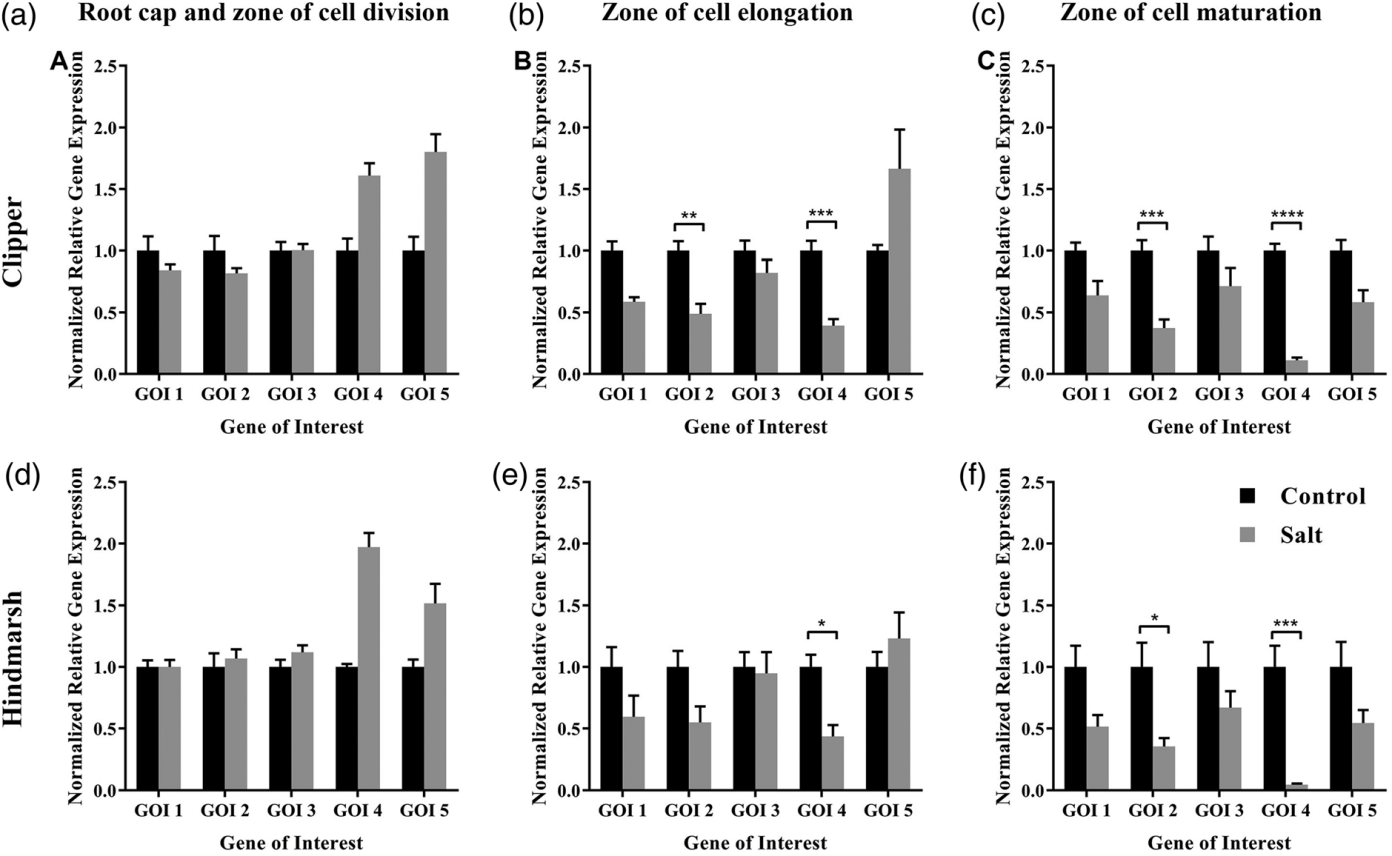
Relative expression levels of HORVU2Hr1G122470 (GOI 1), HORVU3Hr1G023960 (GOI 2), HORVU5Hr1G084740 (GOI 3), HORVU3Hr1G079900 (GOI 4), and HORVU4Hr1G088470 (GOI 5) in three developmental root zones of seminal roots of barley cultivars Clipper and Hindmarsh under control and salt (150 mM of NaCl) conditions. (a) Root cap and zone of cell division (S1)—Clipper, (b) Zone of cell elongation (S2)—Clipper, (c) Zone of cell maturation (S3)—Clipper, (d) Root cap and zone of cell division (S1)—Hindmarsh, (e) Zone of cell elongation (S2)—Hindmarsh, (f) Zone of cell maturation (S3)—Hindmarsh. ^*^ *P* < .05, ^**^ *P* < .01, ^***^ *P* < .001, ^****^ *P* < .0001. GOI, gene of interest

After 48 h under 150 mM of NaCl, there were no significant differences in the relative gene expression in any of the GOI in S1 (Figure 3a). However, GOI 2 and GOI 4 showed a significant (*P* < .05) downregulation in their relative levels of expression of −2.04‐ and −2.54‐fold in S2 (Figure 3b), respectively. Further, there was a significant (*P* < .05) downregulation in the relative levels of expression of GOI 2 and GOI 4 of −2.67‐ and −9.01‐fold in S3 (Figure 3c), respectively.

#### 3.4.2 Changes in the relative gene expression in sections of seminal roots of Hindmarsh grown under control and salt‐treated (150 mM of NaCl) conditions.

Similarly to Clipper, S1 of Hindmarsh roots did not show a significant difference in the levels of any of the five GOI after exposure to a short‐term salt stress (Figure 3d). In S2, only GOI 4 showed a significant (*P* < .05) downregulation of −2.82‐fold after 48 h of exposure to 150 mM of NaCl (Figure 3E). Whereas S3 of Hindmarsh showed a significant (*P* < .05) change in the relative gene expression of GOI 2 and GOI 4 with a −2.82‐ and −21.74‐fold downregulation, respectively (Figure 3F).

### 3.5 Elemental analysis of barley roots

It is worth noting that we previously released MALDI‐MSI and ICP‐MS data relating to Hindmarsh roots in (Sarabia *et al*., 2018a). These results are presented in this study to present a coherent story regarding the effects of a short‐term salt stress in seminal roots of barley.

**Figure 4:**
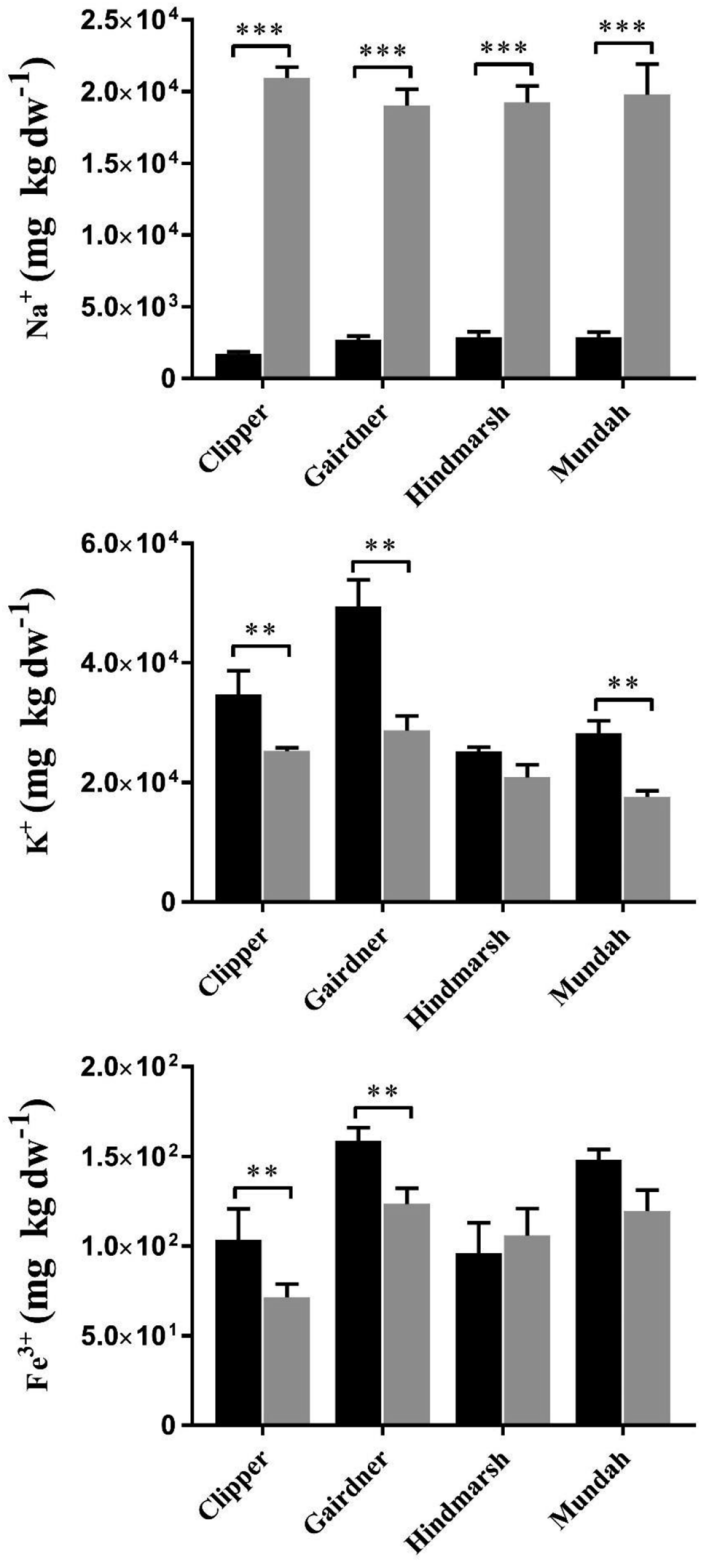
Mean Sodium, Potassium and Iron concentrations ± SE (n = 3) in root tissues from four barley cultivars grown under control and salt (150 mM NaCl) conditions. Black bar: control, grey bar: salt.Asterisks indicate significant difference at p < 0.05, ** p <0.01, p *** < 0.001 based on paired Student's t test

We measured the levels of K^+^, Na^+^, P^+^, Mg^2+^, Ca^2+^, Fe^3+^, Zn^2+^, Mn^2+^, and Cu^2+^ in seminal roots of control and salt‐stressed barley seedlings using ICP‐MS (Table S8, Figure 4). All barley cultivars showed changes in the total content of K, Na, and Fe following a short‐term salinity stress (Figure 4). Potassium was significantly reduced after salinity stress in Clipper, Gairdner, and Mundah (*P* < .001) with a −1.38‐fold, −1.72‐fold and −1.60‐fold reduction, respectively. Hindmarsh showed a non‐significant reduction of −1.21‐fold in the K^+^ content after a short‐term salt stress. There was a significant increase in the levels of Na^+^ among the four barley cultivars with Clipper showing the highest accumulation of Na followed by Gairdner, Mundah, and Hindmarsh with a 12.23‐fold, 7.08‐fold, 6.89‐fold, and 6.66‐fold increase in Na concentration after a short‐term salt stress, respectively. Additionally, the Fe concentration was significantly altered in Clipper and Gairdner roots with a −1.45‐fold and a −1.28‐fold reduction after a short‐term salt stress, respectively. There were no significant changes in the concentration of P, Mg, Ca, Zn, Mn, and Cu in roots of the four barley cultivars after salt stress.

Under control conditions, Clipper and Gairdner had the highest K^+^/Na^+^ ratio with 20.23 ± 1.27 and 18.43 ± 0.40, respectively. By contrast, Gairdner exhibited the highest K^+^/Na^+^ ratio (1.50 ± 0.40), and Hindmarsh exhibited the lowest K^+^/Na^+^ ratio (1.08 ± 0.08) following a short‐term salt stress. Regarding the overall fold‐change in K^+^/Na^+^ ratio after salt treatment, Clipper was the most affected cultivar and Mundah the least affected with a −16.7‐fold and −5.9‐fold reduction, respectively (Table 1).

**Table 1:** K^+^/Na^+^ ratios of seminal roots in control (C) and salt (S) conditions in four barley cultivars as determined by inductively coupled plasma mass spectrometry

Cultivars	Treatment	K^+^/Na^+^
Clipper	C	20.23 ± 1.27
	S	1.21 ± 0.04
Gairdner	C	18.43 ± 0.40
	S	1.50 ± 0.40
Hindmarsh	C	8.99 ± 1.86
	S	1.08 ± 0.08
Mundah	C	7.31 ± 1.11
	S	1.23 ± 0.07

## 4 DISCUSSION

### 4.1 Impact of a short‐term salt stress on barley seminal root growth on agar media

Differences in seminal root development (Figure S1) of the four barley cultivars can partly be explained as a result of differences in early vigour among the barley seeds. Early vigour is a combination of the ability of the seed to uniformly germinate and emerge after planting and the ability of the young plant to grow and develop after emergence (Namuco, Cairns, & Johnson, 2009). Additionally, it is worth noting that a short‐term salt stress caused a slower root development among all four barley cultivars (Figure S1). Thus, Gairdner maintained the longest total seminal root length, and Clipper showed the most significant reduction in total seminal root length. A reduction in root growth after exposure to salt stress is associated with an inhibition of cell division and cell expansion, as previously reported in barley (Shelden et al., 2013; Tabur & Demir, 2009; Tabur & Demir, 2010).

Maintenance of root growth rates under abiotic stress can ensure that plants get enough nutrients from soil and thus resulting in better chances of survival under prolonged exposure to salt conditions. Thus, Gairdner and Hindmarsh were the more tolerant cultivars to a short‐term salt stress by maintaining a higher level of seminal root formation compared with Mundah and Clipper.

### 4.2 Changes in the lipid profiles of three developmental root zones in response to a short‐term salt stress

Plants' responses to abiotic stresses occur in a tissue and cell‐specific manner because plant biochemistry and physiology are spatially differentiated at a cellular and subcellular levels (Sumner, L. W., 2010). There is a highly specialized biological function across different developmental root zones in seminal roots of barley with specific root‐zone responses of both metabolites and transcripts (Hill et al., 2016; Shelden et al., 2016). Thus, in order to elucidate the role of lipid species in the salinity response that occurs in roots following a short‐term salt stress, we analysed the changes in the lipid profiles of three developmental root zones of barley plants using LC‐MS lipidomics and MALDI‐MSI.

#### 4.2.1 Changes in glycerophospholipids support increased membrane lipid synthesis suggesting PC modulation may play a role to confer an adaptive advantage to salt stress

There were significant increases in the levels of PC, PE, PG, PI, and PS species in S1 of Clipper and Mundah, and in S2 of Gairdner. Whereas, there was a significant reduction in the levels of LPC, LPE, LPI, LPG, and LPS in S1 of Hindmarsh and Mundah; in S2 of Clipper Hindmarsh and Mundah; and in S3 of Gairdner, Hindmarsh, and Mundah and a decrease in PC, PE, PG, PI and PS of the 36:n family in S3 of Gairdner, Hindmarsh, and Mundah (Figure 2). Increased levels of GPs in response to abiotic stresses are well documented and are thought to play important roles in membrane remodelling by modulation of membrane fluidity and maintenance of osmotic balance during hyperosmotic stress conditions imposed due to high salinity (Mansour, 2013).

Phosphatidylcholine is one of the major GP classes that was altered in response to osmotic stress imposed by salinity showing a significant upregulation in S1 of both Clipper and Gairdner and a significant downregulation in S3 of Gairdner, Hindmarsh, and Mundah (Figure 2). PCs are important lipids contributing to membrane structure and function involved in adaptive responses to abiotic stresses through changes in their concentration (Tasseva, Richard, & Zachowski, 2004). Increases in PC species following salt stress have been found in salt‐tolerant plants including cultures of *Spartina patens* (Wu, Seliskar, & Gallagher, 2005) and *Catharanthus roseus* (Elkahoui, Smaoui, Zarrouk, Ghrir, & Limam, 2004), as well as epidermal bladder cells of *Mesembryanthemum crystallinum* (Barkla, Garibay‐Hernandez, Melzer, Rupasinghe, & Roessner, 2018). By contrast, decreases in PC species have been observed in more salt‐sensitive plants such as oats and wheat (Magdy, Mansour, Hasselt, & Kuiper, 1994; Norberg & Liljenberg, 1991); this may suggest that a plant's ability to increase or maintain PC levels is an adaptive mechanism for salt tolerance.

Maintenance and increase of highly unsaturated PC species is considered to potentially be linked to increased membrane fluidity (Van Meer, Voelker, & Feigenson, 2008). Our study suggests that PC species containing a higher number of unsaturations are preferentially maintained after salt stress. This may be due to the synthesis of higher levels of these lipid species in S1 of Clipper and Mundah or by preserving the levels of the most unsaturated lipids in S3 of Gairdner and Hindmarsh (Figure 2).

CL are structural phospholipids predominantly found in the mitochondria (Darwish, Testerink, Khalil, El‐Shihy, & Munnik, 2009; Pan, Jones, & Hu, 2014). In the salt‐sensitive*Arabidopsis*, CLs have been described to play a role in mitochondrial fission by stabilizing the protein complex of DINAMIN‐Related Protein 3 during environmental stress (Pan et al., 2014). In crops, the role of CL remains unclear, as two studies in rice and barley have only reported decreases in CL levels following salt stress (Darwish et al., 2009; Meringer et al., 2016). In our study, the levels of most of the analysed CL species remained unchanged after salt stress (Figure S2).

#### 4.2.2 Changes in neutral glycerolipids following salt‐stress

A decline in the concentration of MGDG and DGDG lipid species has been reported in response to abiotic stresses in sensitive species (Bejaoui et al., 2016; Djebali et al., 2005; Gigon, Matos, Laffray, Zuily‐Fodil, & Pham‐Thi,^2004^; Moellering, Muthan, & Benning, 2010; Monteiro de Paula et al., 1993). A small number of studies have found non‐uniform changes in roots of several crops in response to salt stress with barley and salt‐sensitive wheat not showing a significant effect (Brown & Dupont, 1989; Magdy et al., 1994), whereas a salt‐tolerant maize cultivar shows a decrease in MGDG and DGDG content following salt stress (Salama, Mansour, Ali, & Abou‐Hadid,^2007^). In our study, most of the analysed MGDG and DGDG species, besides those belonging to the 36:n family, were not significantly changed following a short‐term salt stress (**Figure** **S2**). These results show that more research is needed to elucidate the role of MGDG and DGDG species in the response of roots to salt stress before drawing a conclusion.

DAG is known to be an intermediate in the synthesis of membrane lipids and is involved in phospholipid signalling in plant cells (Dong, Lv, Xia, & Wang, 2012). Phosphorylation of DAG by DAG kinases leads to conversion into PA that is further transformed into more complex GP (i.e., PC and PE) in response to salinity stress (Munnik & Testerink, 2009; Ruelland et al., 2015). Neutral glycerolipids, such as DAG of the 36:n family, were highly responsive in the S3 of Gairdner, Hindmarsh, and Mundah showing a marked decrease in their concentration (Figure 1) following a short‐term salt stress. This suggests that DAG containing 16:n and 18:n fatty acids may have been repurposed for the synthesis or maintenance of GP (i.e., PE and PG) in S3 of Gairdner, Hindmarsh, and Mundah that also had significant decreases in PC and PI levels (Figure 1).

#### 4.2.3 Sphingolipid and sterol changes in response to salt stress

Sterols serve as regulators for membrane fluidity and permeability (Wang, Juliani, Jespersen, & Huang, 2017). Increased levels in sterol contents have been positively linked to abiotic stress tolerance in cold stress for potatoes and salt stress for tomatoes (Kerkeb, Donaire, Venema, & Rodríguez‐Rosales,^2001^; Palta, Whitaker, & Weiss, 1993). Additionally, Cers are sphingolipid species involved in important signalling events such as programmed cell death (PCD) in plants grown under adverse environmental conditions (Liang et al., 2003). However, Cer involvement in PCD was reported to be directly dependant on the modifications exhibited by their very long chain fatty acid (VLCFA) moiety (Townley, McDonald, Jenkins, Knight, & Leaver, 2005). Increased levels of non‐hydroxylatedCer‐induced PCD in *Arabidopsis* cells, whereas increased levels of Cer‐OH did not induce PCD (Townley et al., 2005). Further, it was also suggested that hydroxylation of the VLCFA moiety of ceramides is important for the biosynthesis of more complex sphingolipids [i.e., glucosylceramide (GlcCer); Konig et al., 2012].

GlcCer have been implicated in conferring stability to plant cell membranes exposed to drought and cold stress (Kawaguchi, Imai, Naoe, Yasui, & Ohnishi, 2000; Norberg, Månsson, & Liljenberg, 1991), with the GlcCer content reduced following cold acclimation (Uemura, Joseph, & Steponkus, 1995; Uemura & Steponkus, 1994). In a recent study, it has suggested the enhancement in salt tolerance in *Arabidopsis* is due to the overexpression of the ceramide‐catalysing enzyme (*AtACER*) (Wu et al., 2015). However, the exact mechanism by which *AtACER* enhances salt tolerance in *Arabidopsis* remains unknown.

Despite the limited knowledge about the specific role of sphingolipid species in plants exposed to abiotic stresses, one of the hypotheses on sphingolipids involvement in abiotic stress response is related to maintenance of the plant endomembrane system (De Bigault Du Granrut & Cacas, 2016). The endomembrane system is considered as a key plant component in salt stress tolerance, which may be linked to VLCFA‐containing sphingolipids (i.e., glucosylceramides). GlcCer can cluster with sterols forming dynamic microdomains located in the plasma membrane (Cacas et al., 2012). These GlcCer–sterol clusters can work as protein‐sorting mediators either by exerting a chaperon‐like activity that stabilizes the structure of membrane cargo proteins between the Golgi apparatus and the plasma membrane, or by imposing a positive curvature to the membranes facilitating vesicle fusion (Molino et al., 2014).

Mundah was the only cultivar that showed a significant increase in the levels of ASG, Cer‐OH, and hydroxylated glucosylceramides (GlcCer‐OH) in S1, S2, and S3 and a decrease in non‐hydroxylatedGlcCer‐OH in S2 and S3 in response to a short‐term salt stress. By contrast, levels of Cer‐OH,GlcCer‐OH, and ASG were slightly increased in Clipper, Gairdner, and Hindmarsh in S1, S2, and S3 (Figure 1).

In summary, our results suggest that Cer‐OH,GlcCer‐OH, and sterols may be involved in maintenance of membrane stability. First, we consider that an increase or maintenance of the levels of Cer‐OH prevents induction of PCD allowing barley roots to grow under saline conditions. Secondly, Cer‐OH may also be used as substrate for the synthesis of the more complex GlcCer‐OH. Lastly, it is hypothesized that the maintenance and/or increase in the levels of hydroxylated GlcCer and levels of sterols may suggest their involvement in the formation of dynamic microdomains in the plasma membrane thus conferring membrane stability to roots of barley seedlings exposed to high salinity. It is worth noting that significant increases in the concentrations of these lipid species in Mundah suggest that the described reactions are more prevalent in this cultivar in order to facilitate root development when grown under salt conditions.

#### 4.2.4 *De novo* lipid biosynthesis may be involved in maintenance of high levels of GP in barley roots after salt stress

Crosstalk between GP membrane lipids and neutral lipids has been reported and thought to play a role in abiotic stress tolerance (Barkla et al., 2018; Bates, 2016; Péter et al., 2017). In our study, we inferred a great demand for GP to maintain cell development under salt stress from interpretation of the observed decrease in LysoGP and DAG and the increase in GP species across four barley cultivars, especially in S1 of Clipper and Mundah as these cultivars showed a more significant root growth suppression as compared with Hindmarsh and Gairdner.

Further, maintenance of the levels of C16:0, C18:3, C18:2, and C18:1 in barley roots may indicate *de novo* synthesis of FA contributing to the increased levels of GP in salt‐treated barley roots (**Figure** **S3**). This is also supported by the increase in the levels of 34:n lipid species of the PC, PE, and PI (**Figure** **2**) that are mostly made of 16:0_18:1, 16:0_18:2, and 16:0_18:3 FA moieties from *de novo* FA biosynthesis in the plastid (Koo, Ohlrogge, & Pollard, 2004; Li‐Beisson et al., 2013). This supports our results that show an increase in 18:1, 18:2, and 18:3 containing GP species in salt‐treated seminal roots, inferred from the increase or preservation of highly unsaturated lipids belonging to the 32:n, 34:n, 36:n, and 38:n molecular families **(Figure** **2** **)**.

**Figure 5:**
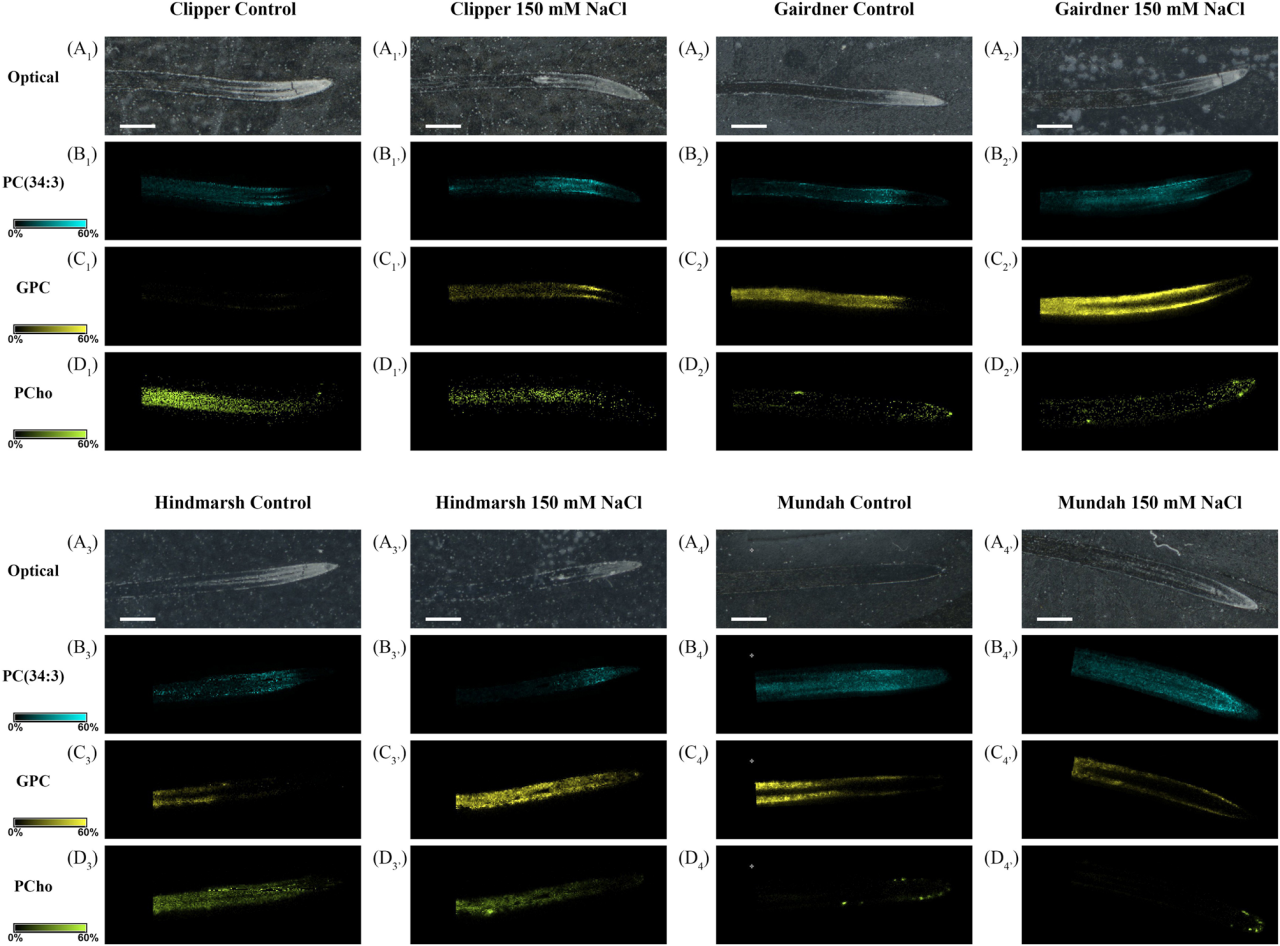
Matrix‐assisted laser desorption/ionization mass spectrometry images of choline derivative compounds found distributed in longitudinal root sections of four barley cultivars grown under control and salt conditions. Ion image are displayed using the same intensity scale for each individual lipid species (solid colour: 0–60%). The mass accuracy was set at <0.005 ppm. Scale bars: 1,000 μm. Control and salt‐treated images were obtained in positive ionization mode with a scanning step size of 30 μm × 30 μm. Mass spectrometry images of the Hindmarsh cultivar have been reproduced from (Sarabia et al., 2018a). Letters: A, optical images; B, PC(34:3); C, GPC; D, PCho. Numbers: 1, Clipper; 2, Gairdner; 3, Hindmarsh; 4, Mundah. GPC: glycerophosphocholine; PCho: phosphocholine [Colour figure can be viewed at http://wileyonlinelibrary.com]

GPC is a biomolecule that showed an increased concentration in seminal roots after salt stress (Figure 5), which is reported to accumulate in response to abiotic stress conditions due to membrane turnover or degradation (Aubert et al., 1996; Menegus & Fronza, 1985; Roscher, Emsley, Raymond, & Roby, 1998). There were several PC, PI, and PS species of the 36:n family with a significant decrease in S3 of Gairdner, Hindmarsh, and Mundah, whereas most of the GP species showed an increase or maintenance in their levels in S1, S2, and S3 of all four cultivars after salt stress (Figure 2). Our results suggest that GPC may act as an intermediate in the synthesis of membrane GP species and also helps stabilize membranes, provide protein structure, and function during hyperosmotic stress (Kwon et al., 1995; Popova & Busheva, 2001), especially in S3 for Gairdner, Hindmarsh, and Mundah where a reduction of some GP species (i.e., PC 36:n) was observed (**Figure** **2**).

### 4.3 Relative gene expression analysis of five genes involved in GP metabolism in response to a short‐term salt stress.

Accumulation of GPC and PC species across root tissues of barley seedlings was one of the most remarkable results found using MALDI‐MSI and LC‐MS/MS. Thus, in order to provide more evidence supporting these results, we analysed the relative gene expression of five genes involved in PC metabolism.

#### 4.3.1 Changes in the relative gene expression of a phosphatidylserine decarboxylase encoding gene

Phosphatidylserine decarboxylase (PSD) is a crucial enzyme catalysing the production of PE from phosphatidylserine (Larsson, Nystrom, & Liljenberg, 2006; Nerlich, von Orlow, Rontein, Hanson, & Dormann, 2007). In this study, a gene encoding a putative PSD (HORVU5Hr1G084740) enzyme did not show significant changes in its levels of expression in two barley cultivars. Thus, these results suggest that the basal rate of conversion of PS to PE is not affected after a short‐term salt stress.

#### 4.3.2 Relative expression of a transcript showing phospholipase D delta activity supports maintenance of basal conversion of PC to PA

Phospholipase D (PLD) are a family of enzymes that hydrolase the phosphodiester bond on the head group of glycerophospholipids producing PA and a soluble head group (Hong et al., 2016). PLDδ activity in *Arabidopsis* has been reported in response to dehydration (Katagiri, Takahashi, & Shinozaki, 2001), freezing tolerance, (Li, Li, Zhang, Welti, & Wang, 2004), H_2_O_2_‐induced cell death (Zhang et al., 2003), under salt stress conditions (Bargmann et al., 2008; Katagiri et al., 2001), basal defence to powdery mildew fungi (Pinosa et al., 2013), and to microtubule‐plasma membrane stabilization under salt stress (Angelini et al., 2018).

In this study, maintenance of the relative gene expression of a putative PLD delta (HORVU5Hr1G084740) suggested that degradation of PC to PA by this putative protein was not significantly altered after a short‐term salt stress. These findings agree with the lipidomics results, which did not show a discriminative increase in PA levels in barley roots after salt stress, maintaining the basal levels of PC to PA conversion (Tables S2, S3, and S4).

#### 4.3.3 Changes in transcripts showing lysophospholipase 2 activity may suggest LPC recycling following salt stress

Lysophospholipases (LysoPLs) are a group of enzymes responsible for hydrolysis of the ester bonds on lysophospholipids releasing free fatty acyl groups and glycerophosphodiester derivatives (Gao, Li, Xiao, & Chye, 2010; Kim et al., 2000). In this study, two transcripts encoding for a putative LysoPL A2 (HORVU2Hr1G122470) and a putative LysoPL A‐like (HORVU3Hr1G023960) enzyme showed a downregulation in their levels of expression under saline conditions. These results suggest that lysophospholipids do not undergo deacylation of their fatty acyl chain to form GPC.

#### 4.3.4 Downregulation of a transcript showing glycerophosphodiester phosphodiesterase activity supports accumulation of GPC in roots after salt stress

Glycerophosphodiester phosphodiesterase (GDPD) are a family of evolutionarily conserved hydrolase enzymes that catabolize the breakdown of a range of glycerophosphodiesters into *sn*‐glycerol‐3‐phosphate and alcohol (Kim, Hong, Jang, & Seo, 2012). GDPD activity has been reported to be significantly upregulated under inorganic phosphate starvation to maintain phosphate homeostasis in *Arabidopsis* (Cheng, Y *et al*., 2011), rice (Jeong et al., 2017; Mehra & Giri, 2016; Mehra, Pandey, Verma, & Giri, 2019), oats (Andersson, Larsson, Tjellström, Liljenberg, & Sandelius, 2005), maize (Calderon‐Vazquez, Ibarra‐Laclette,Caballero‐Perez, & Herrera‐Estrella,^2008^), chickpeas (Mehra & Giri, 2016), white lupin (Cheng, L *et al*., 2011), and barley (Ren et al., 2018).

In this study, a gene encoding for a putative GDPD2 (HORVU3Hr1G079900) enzyme showed a significant downregulation of its relative expression in barley roots after salt stress. Our results directly demonstrate that an accumulation of GPC when combined with a downregulation of the putative GDPD2 (HORVU3Hr1G079900) suggests that GPC is not being degraded to *sn*‐glycerol‐3‐phosphate and choline in barley roots after salt stress.

#### 4.3.5 Phosphatidylcholine recycling in response to a short‐term salt stress

**Figure 6:**
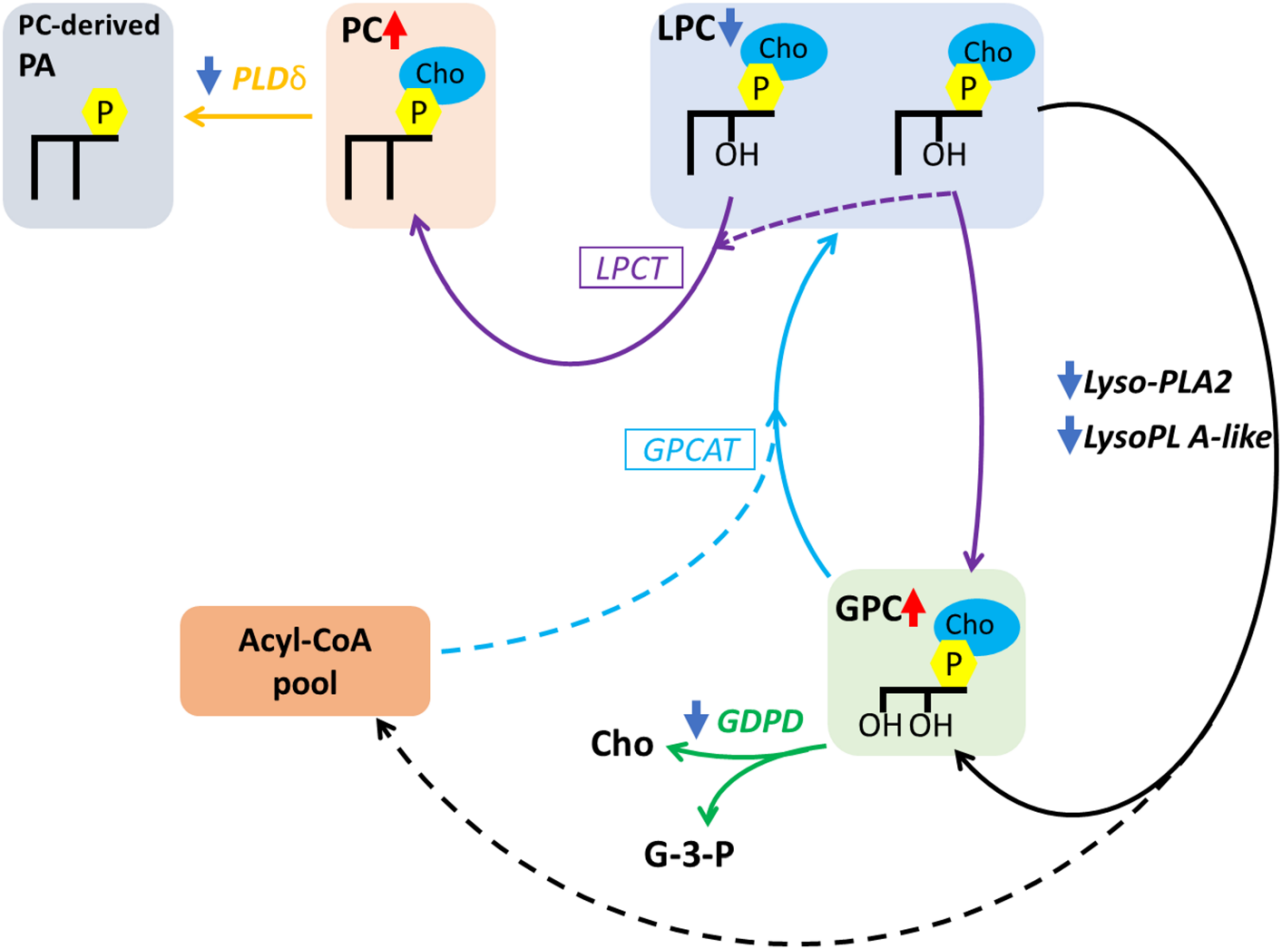
Proposed metabolic modifications to phosphatidylcholine and its derivatives in seminal roots of barley following a short‐term salt stress. The hypothesised reactions involved in PC maintenance. Solid lines: flux of the glycerol backbone. Dashed lines: acyl fluxes. Italized names are enzymatic reactions. Boxed enzymes are those of which the encoding gene is still unclear. Non‐italized are lipid names. Colour schemes: Black, Lyso‐PL; Gold, PLD;Turquoise, GPCAT; Purple, LPCT. Colour arrow: dark red, upregulation/increase;dark blue, downregulation/decrease. Abbreviations: PA – phosphatidic acid; PC – phosphatidylcholine; LPC –lysophosphatidylcholine; GPC – glycerophosphocholine; Cho – choline; G‐3‐P ‐glycerol‐3‐phosphate. PLDδ – phospholipase D delta; LPCT –lysophosphatidylcholine transacylase; GPCAT – glycerophosphocholine:acyl‐CoAacyltransferase; LysoPL A2 – lysophospholipase A2; GDPD ‐ glycerophosphodiesterphosphodiesterase. *Image was adapted from (Bates, 2016). [Colour figure can be viewed at http://wileyonlinelibrary.com]

In this study, genes encoding for three putative enzymes (LysoPL A2, LysoPL‐like, and GDPD) involved in PC maintenance have shown downregulation in roots of two barley cultivars (Figure 6). These results agree with the lipidomics findings of an accumulation of PC species, decrease of LPC species, and accumulation of GPC across three developmental root tissues following a short‐term salt stress (Tables S2, S3, and S4, Figure 5). To account for these observations, we hypothesize that the increase and maintenance of the levels of PC species after salt stress may be a result of the combined activity of GPC:acyl‐CoA acyltransferase (GPCAT; Stalberg, Neal, Ronne, & Stahl, 2008) and LPC transacylase (LPCT; Lager et al., 2015). In this mechanism, GPCAT mediates GPC acylation with acyl‐CoA at both *sn* positions forming LPC that is the followed by LPCT transacylation of the acyl group of one LPC molecule to a second LPC molecule resulting in the generation of PC and GPC (Lager et al., 2015).

LPCT activity has been hypothesized to be present due to the significant increase in the levels of PC, decrease in the levels of LPC species and a downregulation of expression of two genes encoding LysoPL A2‐related enzymes. The latter also provided evidence that GPC accumulation may be the result of LPCT‐mediated transacylation reactions instead of LysoPL A2‐mediated deacylation reactions. Further, GPC produced by LPCT activity was hypothesized to be the substrate for resynthesis of PC via the GPCAT pathway.

It is worth noting that little is known about the physiological role of phosphodiesters, such as GPC, in plant systems in response to salt stress. In contrast, GPC has been hypothesized to serve as a compatible organic osmolyte to protect against the high levels of NaCl in renal cells in mammals (Gallazzini & Burg, 2009). Because this study strongly supports GPC accumulation in response to salinity, further research is necessary to elucidate the roles of GPC in cereals crops besides its involvement in PC resynthesis.

In conclusion, this study shows that barley roots respond to a hyperosmotic stress by altering to levels of lipid species in a tissue‐specific manner. Increases in Cer‐OH in Mundah and maintenance of Cer‐OH in Clipper, Gairdner, and Hindmarsh prevented activation of PCD, whereas decreases in LysoGP and DAG coupled with increases in GP and GPC allowed maintenance of membrane fluidity and stability in all four cultivars. However, increases in GP were more predominant in S1 of Clipper and Mundah that showed higher root growth suppression among all the barley cultivars, thus, necessitating a greater synthesis of GP species in order to facilitate and aid in root development under salt stress.

Additionally, accumulation of GPC in response to salt stress was hypothesized to serve as both an osmolyte and as substrate for the resynthesis of PC. The latter was described to be the result of the combined activity of GPCAT and LPCT enzymes that may confer barley plants an enhanced tolerance to salt stress. The positive role of GPC opens a new avenue for understanding salinity stress response in crops.

## AUTHORSHIP

L.D.S. performed laboratory analyses, analysed the data, and wrote the manuscript. D.L.C. performed ICP‐MS analysis and revised the manuscript. B.A.B., C.B.H., T.R., and U.R. supervised the study and revised the manuscript.

## CONFLICT OF INTEREST

The authors declare no conflict of interest.

## Supporting information


**Data S1.** Supporting information

**Data S2.** Supporting information

**Data S3.** Supporting information

**Table S1.** Length of dissected root sections from barley seminal roots.
S1: root cap and zone of cell division; S2: zone of cell elongation and S3: zone of cell maturation.
**Table S2.** AUC values for discriminative lipid species found between whole root sections of control and salt‐treated barley roots.
**Table S3.** AUC values for discriminative lipid species found between root cap and zone of cell division (S1) root sections of control and salt‐treated barley roots.
**Table S4.** AUC values for discriminative lipid species found between root cap and zone of cell elongation (S2) root sections of control and salt‐treated barley roots.
**Table S5.** AUC values for discriminative lipid species found between root cap and zone of cell maturation (S3) root sections of control and salt‐treated barley roots.
**Table S6.** Description of candidate genes of interest (GOI) chosen from the Glycerophospholipid Metabolism KEGG Pathway (https://www.genome.jp/kegg/pathway/map/map 00564.html).
**Table S7.** Selected candidate target and reference genes, primers, and amplicon characteristics.
**Table S8.** The elemental composition of roots of four barley cultivars grown under control and salt (150 mM of NaCl) conditions.

**Figure S1.** Supporting information

**Figure S2.** Supporting information

**Figure S3.** Supporting information
