# Supplementary material for: Comparative spatial lipidomics analysis reveals cellular lipid remodelling in different developmental zones of barley roots in response to salinity
Source: Plant Cell Environ. 2019 Nov 29;43(2):327–43. doi: 10.1111/pce.13653 (PMC7063987; doi:10.1111/pce.13653)
Supplement: Supplementary file 1 — Data S1. Supporting information [file PCE-43-327-s001.docx]

**Supplementary Materials and Methods**

**Chemicals**

Solvents were purchased from Merck Millipore (Bayswater, VIC, Australia). Chemicals including 2,5-dihydroxy benzoic acid, ethidium bromide, elemental red phosphorus, Supra pure® nitric acid (70%), hydrochloric acid (30%), and blue polypropylene pellet pestles and primers were purchased from Sigma-Aldrich (Castle Hill, NSW, Australia). 1,8-Bis-(l-pyrrolidinyl)naphthalene (BPYN) was synthesised as described in (Alder *et al.*, 1981). Embedding and freezing supplies including cryofilm fitting tool, embedding medium (SCEM), cryofilm 2C(9) (2.0 cm in width) were purchased from Section-Lab Co. Ltd. (Tokyo, Japan). Sectioning supplies including Menzel-Gläser Superfrost Ultra Plus Glass slides, Optimal Cutting Temperature (O.C.T.) compound and Feather® C35 tungsten microtome blades were purchased from Grale HDS (Ringwood, Australia). Lysing Matrix Tubes with 0.5 g Lysing Matrix D (1.4 mm ceramic spheres) were purchased from MP Biomedicals (Seven Hills, NSW, Australia). Elemental standards were purchased from PerkinElmer (Melbourne, VIC, Australia). MyTaq™ Red DNA Polymerase and SensiFAST™ SYBR® No_ROX Kit were purchased from Bioline (NSW, Australia). RNeasy Plant Mini Kit and RNase-Free DNase Set were purchased from Qiagen Pty Ltd (VIC, Australia). SuperScript™ III Reverse Transcriptase, RNaseZap™ and RNaseOUT™ were purchased from Thermo Fisher Scientific (VIC, Australia). Oligo(dT)15 Primer and Set of dATP, dCTP, dGTP and dTTP were purchased from Promega (VIC, Australia).

**Matrix deposition for MALDI-MSI analysis**

2,5-Dihydroxy benzoic acid (2,5-DHB, 50 mg/mL solution in 100% acetone) or 1,8-Bis-(l-pyrrolidinyl)naphthalene (BPYN, 5 mg/mL solution in 100% acetone) was deposited using a HTX TM-Sprayer™ (HTX Industries, Chapel Hill, HC, USA) fitted with a Shimadzu LC20-AD HPLC pump (Shimadzu Australia, Rydalmere, NSW, Australia) with the following settings: solvent system: 100% (v) acetone; Solvent flow rate: 150 µL/min; Nitrogen gas pressure: 10-12 Bar; Nozzle spray temperature: 30° C; Nozzle spray velocity: 1300 mm/min; Spray conditions: 4 passes for DHB and 8 passes for BPYN, spacing of 2 mm with alternate passes at a 90° offset and repeat passes set to an offset of 1 mm.

**Mass spectrometry imaging**

MALDI-MSI analysis was performed using a Bruker (Bruker Daltonics, Bremen, Germany) SolariX XR 7 Tesla Hybrid ESI/MALDI-FT-ICR-MS equipped with a SmartBeam II UV laser using a modified previously described method (Sarabia *et al.*, 2018). The instrument was operated in both positive and negative ion modes using optimized instrumental settings. For full scan experiments, a mass range from *m/z* 100 – 2100 was selected with the instrument set to broadband mode with a time domain for acquisition of 2M providing an estimated resolving power of 130,000 at *m/z* 400. The laser was set to 35% – 40% power using a minimum spot size resulting in optimal ablation of the sample. A total of 250 – 350 laser shots were fired per spectrum at a frequency of 2 kHz within a 30 µm × 30 µm array. Optical images of the matrix coated root sections were acquired using an Epson Perfection 4870 Photo flatbed PC scanner (Epson Australia, NSW, Australia) at 4,800 dpi resolution.

**Alder RW, Bryce MR, Goode NC, Miller N, Owen J. 1981.** Preparation of a range of NNN′N′-tetrasubstituted 1,8-diaminonaphthalenes. *Journal of the Chemical Society, Perkin Transactions 1*(0): 2840-2847.

**Sarabia LD, Boughton BA, Rupasinghe T, van de Meene AML, Callahan DL, Hill CB, Roessner U. 2018.** High-mass-resolution MALDI mass spectrometry imaging reveals detailed spatial distribution of metabolites and lipids in roots of barley seedlings in response to salinity stress. *Metabolomics* **14**(5): 63.
