# Supplementary material for: Comparative spatial lipidomics analysis reveals cellular lipid remodelling in different developmental zones of barley roots in response to salinity
Source: Plant Cell Environ. 2019 Nov 29;43(2):327–43. doi: 10.1111/pce.13653 (PMC7063987; doi:10.1111/pce.13653)
Supplement: Supplementary file 4 — Table S1. Length of dissected root sections from barley seminal roots. S1: root cap and zone of cell division; S2: zone of cell elongation and S3: zone of cell maturation. Table S2. AUC values for discriminative lipid species found between whole root sections of control and salt‐treated barley roots. Table S3. AUC values for discriminative lipid species found between root cap and zone of cell division (S1) root sections of control and salt‐treated barley roots. Table S4. AUC values for discriminative lipid species found between root cap and zone of cell elongation (S2) root sections of control and salt‐treated barley roots. Table S5. AUC values for discriminative lipid species found between root cap and zone of cell maturation (S3) root sections of control and salt‐treated barley roots. Table S6. Description of candidate genes of interest (GOI) chosen from the Glycerophospholipid Metabolism KEGG Pathway (https://www.genome.jp/kegg/pathway/map/map 00564.html). Table S7. Selected candidate target and reference genes, primers, and amplicon characteristics. Table S8. The elemental composition of roots of four barley cultivars grown under control and salt (150 mM of NaCl) conditions. [file PCE-43-327-s004.docx]

**Table S1:** Length of dissected root sections from barley seminal roots.

S1: root cap and zone of cell division; S2: zone of cell elongation and S3: zone of cell maturation.

| **Cultivar** | **Treatment** | **S1 (mm)** | **S2 (mm)** | **S3 (mm)** |
| --- | --- | --- | --- | --- |
| Mundah | Control | 0 – 1.50 | 1.50 – 4.00 | 4.00 – 6.50 |
|  | Salt | 0 – 1.20 | 1.20 – 3.00 | 3.00 – 5.00 |
| Hindmarsh | Control | 0 – 1.25 | 1.25 – 3.75 | 3.75 – 6.25 |
|  | Salt | 0 – 1.25 | 1.25 – 3.25 | 3.25 – 5.25 |
| Gairdner | Control | 0 – 2.00 | 2.00 – 4.50 | 4.50 – 6.50 |
|  | Salt | 0 – 1.75 | 1.75 – 4.00 | 4.00 – 6.25 |
| Clipper | Control | 0 – 1.75 | 1.75 – 4.50 | 4.50 – 6.50 |
|  | Salt | 0 – 1.50 | 1.50 – 2.50 | 2.50 – 3.50 |

**Table S2:** AUC values for discriminative lipid species found between whole root sections of control and salt-treated barley roots.

*AUC > 0.7* the peak was discriminative in control roots. **AUC < 0.3** the peak was discriminative in salt treated roots.

*Lipid tentative annotations were based on accurate precursor mass search (< 5 ppm) against the LIPID MAPS database (Fahy *et al.*, 2007).

| ***m/z*** | **Name** | **Adduct** | **C** | **G** | **H** | **M** |
| --- | --- | --- | --- | --- | --- | --- |
| 235.1651 | 1,13-Dihydroxy-herbertene | [M+H]^+^ | **0.06** | **0.17** | 0.44 | *0.77* |
| 241.0128 | Lipoic acid | [M+Cl]^-^ | 0.56 | 0.55 | *0.74* | 0.57 |
| 255.2334 | Palmitic acid | [M-H]^-^ | 0.55 | 0.53 | *0.74* | 0.51 |
| 275.1257 | Dehydrofalcarinone | [M+Cl]^-^ | 0.54 | 0.53 | *0.75* | 0.54 |
| 277.1158 | (9R,13R)-1a,1b-dihomo-jasmonic acid | [M+K]^+^ | **0.23** | **0.25** | 0.50 | 0.67 |
| 277.2178 | alpha-Linolenic acid | [M-H]^-^ | 0.54 | 0.62 | *0.73* | 0.50 |
| 279.2335 | 10,13-Octadecadienoic acid | [M-H]^-^ | 0.51 | 0.61 | *0.77* | 0.52 |
| 281.2496 | 10-Octadecenoic acid | [M-H]^-^ | 0.53 | 0.58 | *0.78* | 0.48 |
| 283.0924 | Hemigossypol | [M+Na]^+^ | 0.57 | 0.55 | 0.34 | *0.75* |
| 299.0549 | 2'-Hydroxybiochanin A | [M-H]^-^ | 0.50 | 0.53 | *0.70* | 0.51 |
| 309.2809 | 10Z-Eicosenoic acid | [M-H]^-^ | 0.54 | 0.53 | *0.71* | 0.55 |
| 315.0689 | (+)-Bornyl-diphosphate | [M+H]^+^ | **0.03** | **0.09** | 0.47 | 0.52 |
| 331.1992 | 3a,17a-Dihydroxy-5b-androstane | [M+K]^+^ | 0.50 | 0.38 | **0.21** | 0.52 |
| 389.2672 | 1alpha-hydroxy-25,26,27-trinorvitamin D3 24-carboxylic acid | [M+H]^+^ | 0.44 | 0.47 | 0.56 | *0.87* |
| 390.2751 | Anandamide (20:2, n-6) | [M+K]^+^ | 0.46 | 0.53 | 0.50 | *0.81* |
| 397.1707 | 7S,8S-epoxy-17R-HDHA | [M+K]^+^ | 0.39 | **0.20** | **0.30** | 0.53 |
| 398.2620 | Phytosphingosine 1-phosphate | [M+H]^+^ | 0.61 | *0.80* | 0.49 | 0.39 |
| 409.2373 | LPA(16:0) | [M-H]^-^ | 0.49 | 0.53 | *0.72* | 0.53 |
| 409.2563 | 6-deoxyerythronolide B | [M+Na]^+^ | 0.52 | *0.72* | 0.50 | 0.38 |
| 409.2665 | Dioctyl hexanedioate | [M+K]^+^ | 0.52 | *0.71* | 0.45 | 0.49 |
| 413.2106 | Docosatrienoate | [M-H]^-^ | 0.53 | 0.62 | *0.74* | 0.52 |
| 433.1555 | Prebarbigerone | [M+Na]^+^ | **0.07** | 0.26 | 0.42 | 0.56 |
| 465.2633 | Minabeolide-2 | [M-H]^-^ | 0.49 | 0.52 | *0.71* | 0.51 |
| 571.2903 | PI(16:0) | [M-H]^-^ | 0.44 | 0.59 | *0.72* | 0.52 |
| 650.4386 | LPC(25:0(CHO)) | [M+H]^+^ | 0.68 | *0.71* | 0.42 | 0.49 |
| 669.4520 | PA(34:3) | [M-H]^-^ | 0.51 | 0.55 | *0.71* | 0.50 |
| 671.4670 | PA(34:2) | [M-H]^-^ | 0.46 | 0.53 | *0.72* | 0.52 |
| 673.4828 | PA(34:1) | [M-H]^-^ | 0.51 | 0.50 | *0.73* | 0.51 |
| 691.4356 | PA(36:6) | [M-H]^-^ | 0.55 | 0.58 | *0.71* | 0.54 |
| 693.4516 | PA(36:5) | [M-H]^-^ | 0.52 | 0.59 | *0.75* | 0.55 |
| 695.4681 | PA(36:4) | [M-H]^-^ | 0.45 | 0.58 | *0.76* | 0.56 |
| 697.4826 | PA(36:3) | [M-H]^-^ | 0.49 | 0.59 | *0.82* | 0.51 |
| 699.4986 | PA(36:2) | [M-H]^-^ | 0.57 | 0.54 | *0.78* | 0.52 |
| 712.4941 | PE(34:3) | [M-H]^-^ | 0.50 | 0.63 | *0.71* | 0.52 |
| 712.5385 | GlcCer(d14:2/20:0(2OH)) | [M-H]^-^ | 0.55 | 0.64 | *0.71* | 0.54 |
| 714.5096 | PE(34:2) | [M-H]^-^ | 0.43 | 0.62 | *0.73* | 0.57 |
| 723.4996 | PA(38:4) | [M-H]^-^ | 0.57 | 0.53 | *0.72* | 0.53 |
| 743.4887 | PG(34:3) | [M-H]^-^ | 0.46 | 0.59 | *0.76* | 0.51 |
| 745.5043 | PG(34:2) | [M-H]^-^ | 0.40 | 0.55 | *0.77* | 0.52 |
| 747.5203 | PG(34:1) | [M-H]^-^ | 0.50 | 0.48 | *0.70* | 0.50 |
| 752.5071 | PI-Cer(d18:1/14:0) | [M+H]^+^ | *0.89* | 0.62 | 0.52 | 0.68 |
| 798.5405 | PI-Cer(d18:0/16:0(2OH)) | [M+H]^+^ | *0.73* | *0.77* | 0.38 | 0.47 |
| 831.5045 | PI(34:3) | [M-H]^-^ | 0.53 | 0.57 | *0.72* | 0.54 |
| 833.5217 | PI(34:2) | [M-H]^-^ | 0.50 | 0.57 | *0.77* | 0.58 |
| 835.5379 | PI(34:1) | [M-H]^-^ | 0.55 | 0.59 | *0.76* | 0.51 |
| 857.5215 | PI(36:4) | [M-H]^-^ | 0.52 | 0.57 | *0.72* | 0.56 |
| 859.5373 | PI(36:3) | [M-H]^-^ | 0.57 | 0.56 | *0.73* | 0.51 |

* C: Clipper, G: Gairdner, H: Hindmarsh, M: Mundah.

Abbreviations: LPA: lysophosphatidic acid, LPC: lysophosphatidylcholine, LPE: lysophosphatidylethanolamine, PA: phosphatidic acid, PC: phosphatidylcholine, PE: phosphatidylethanolamine, PG: phosphatidylglycerol, PI: phosphatidylinositol, PI-Cer: phosphatidylinositol ceramide

**Table S3:** AUC values for discriminative lipid species found between root cap and zone of cell division (S1) root sections of control and salt-treated barley roots.

*AUC > 0.7* the peak was discriminative in control roots. **AUC < 0.3** the peak was discriminative in salt treated roots.

**Lipid tentative annotations were based on accurate precursor mass search (< 5 ppm) against the LIPID MAPS database (Fahy et al., 2007).*

| ***m/z*** | **Name** | **Adduct** | **C** | **G** | **H** | **M** |
| --- | --- | --- | --- | --- | --- | --- |
| 134.0473 | (+)-threo-2-Amino-3,4-dihydroxybutanoic acid | [M-H]^-^ | **0.21** | 0.52 | 0.54 | 0.49 |
| 145.0619 | 3,4-decadiene-6,8-diyn-1-ol | [M-H]^-^ | **0.05** | *0.88* | 0.50 | 0.56 |
| 161.0456 | 2,3-dimethylmalic acid | [M-H]^-^ | **0.06** | 0.51 | 0.50 | 0.51 |
| 179.0562 | Fuconic acid | [M-H]^-^ | **0.00** | *0.81* | 0.46 | 0.52 |
| 196.0384 | Aminoadipic acid | [M+Cl]^-^ | **0.23** | 0.50 | 0.46 | 0.50 |
| 225.0287 | Nemotinic acid | [M+Cl]^-^ | **0.15** | *0.84* | 0.59 | 0.59 |
| 227.0330 | Mevalonate-P | [M-H]^-^ | 0.45 | 0.54 | *0.72* | 0.65 |
| 235.1651 | 1,13-Dihydroxy-herbertene | [M+H]^+^ | **0.11** | **0.09** | 0.43 | *0.81* |
| 241.0128 | Lipoic acid | [M+Cl]^-^ | *0.71* | 0.59 | *0.77* | *0.74* |
| 243.0630 | Oxyresveratrol | [M-H]^-^ | 0.32 | *0.74* | *0.72* | 0.60 |
| 255.2334 | Palmitic acid | [M-H]^-^ | *0.84* | 0.46 | *0.81* | *0.73* |
| 257.2401 | Decanoylcholine | [M-H]^-^ | *0.70* | 0.50 | 0.69 | 0.51 |
| 275.0597 | Thysanone | [M-H]^-^ | **0.26** | 0.62 | 0.62 | 0.61 |
| 275.1257 | Dehydrofalcarinone | [M+Cl]^-^ | 0.55 | 0.60 | *0.77* | 0.58 |
| 277.1158 | (9R,13R)-1a,1b-dihomo-jasmonic acid | [M+K]^+^ | 0.54 | 0.41 | 0.50 | *0.76* |
| 279.2335 | 10,13-Octadecadienoic acid | [M-H]^-^ | 0.63 | 0.59 | *0.72* | 0.67 |
| 281.2496 | 10-Octadecenoic acid | [M-H]^-^ | 0.64 | 0.56 | *0.86* | 0.61 |
| 283.0924 | Hemigossypol | [M+Na]^+^ | 0.62 | **0.26** | 0.45 | 0.56 |
| 295.2285 | 10-keto-12Z-octadecenoic acid | [M-H]^-^ | *0.77* | **0.28** | *0.81* | *0.72* |
| 299.0549 | 2'-Hydroxybiochanin A | [M-H]^-^ | 0.65 | 0.48 | *0.75* | 0.63 |
| 301.0334 | 6-Hydroxykaempferol | [M-H]^-^ | 0.41 | 0.69 | *0.73* | 0.63 |
| 309.2809 | 10Z-Eicosenoic acid | [M-H]^-^ | 0.55 | 0.55 | *0.74* | 0.63 |
| 315.0689 | (+)-Bornyl-diphosphate | [M+H]^+^ | **0.05** | **0.06** | 0.48 | 0.57 |
| 323.0292 | Carthamidin | [M+Cl]^-^ | 0.51 | *0.76* | *0.81* | 0.66 |
| 325.1262 | Diisopentyl thiomalate | [M+Cl]^-^ | **0.04** | *0.85* | 0.47 | 0.55 |
| 345.1382 | Dihydrokanakugiol | [M-H]^-^ | **0.29** | 0.55 | 0.63 | 0.58 |
| 359.2003 | 1-Acetoxy-2-hydroxy-16-heptadecyn-4-one | [M+Cl]^-^ | **0.29** | 0.61 | *0.74* | 0.52 |
| 361.2332 | 14R,21R-diHDHA | [M+H]^+^ | *0.75* | 0.39 | 0.65 | *0.74* |
| 389.2672 | 1alpha-hydroxy-25,26,27-trinorvitamin D3 24-carboxylic acid | [M+H]^+^ | 0.39 | **0.15** | 0.51 | 0.62 |
| 390.2751 | Anandamide (20:2, n-6) | [M+K]^+^ | 0.44 | **0.19** | 0.50 | 0.65 |
| 397.1707 | 7S,8S-epoxy-17R-HDHA | [M+K]^+^ | 0.45 | **0.27** | 0.39 | 0.50 |
| 399.1443 | 2'-Hydroxypiscerythrinetin | [M+H]^+^ | 0.70 | 0.38 | 0.47 | *0.83* |
| 409.2373 | LPA(16:0) | [M-H]^-^ | *0.77* | 0.46 | *0.80* | 0.70 |
| 409.2665 | Dioctyl hexanedioate | [M+K]^+^ | 0.51 | 0.48 | **0.28** | 0.51 |
| 425.2831 | Sorbitan palmitate | [M+Na]^+^ | 0.53 | 0.41 | *0.73* | 0.48 |
| 433.1555 | Prebarbigerone | [M+Na]^+^ | **0.16** | **0.23** | 0.45 | 0.68 |
| 433.2370 | LPA(18:2) | [M-H]^-^ | 0.52 | *0.75* | 0.64 | *0.83* |
| 435.2527 | LPA(18:1) | [M-H]^-^ | 0.57 | 0.60 | *0.75* | 0.65 |
| 437.1360 | Antiarone K | [M+Cl]^-^ | 0.67 | 0.53 | *0.72* | 0.61 |
| 439.0867 | 9,10-dibromo-stearic acid | [M-H]^-^ | 0.34 | 0.67 | *0.70* | 0.58 |
| 445.2873 | beta-tocotrienol | [M+Cl]^-^ | **0.24** | 0.57 | 0.52 | 0.45 |
| 452.2796 | LPE(16:0) | [M-H]^-^ | *0.78* | 0.51 | *0.75* | 0.63 |
| 465.2633 | Minabeolide-2 | [M-H]^-^ | 0.57 | 0.42 | *0.75* | 0.62 |
| 474.2637 | LPE(18:3) | [M-H]^-^ | 0.43 | *0.80* | 0.52 | *0.79* |
| 476.2794 | LPE(18:2) | [M-H]^-^ | 0.44 | *0.74* | 0.63 | *0.79* |
| 483.2740 | PG(16:0) | [M-H]^-^ | *0.81* | 0.42 | *0.82* | 0.68 |
| 495.1662 | Lupinisoflavone N | [M+Na]^+^ | 0.59 | 0.62 | 0.34 | *0.73* |
| 505.2583 | PG(18:3) | [M-H]^-^ | 0.34 | *0.75* | 0.59 | *0.82* |
| 507.2742 | PG(18:2) | [M-H]^-^ | 0.44 | 0.64 | 0.63 | *0.74* |
| 518.3239 | LPC(18:3) | [M+H]^+^ | **0.26** | 0.43 | 0.31 | 0.32 |
| 520.3394 | LPC(18:2) | [M+H]^+^ | **0.23** | 0.50 | **0.28** | 0.44 |
| 542.3215 | LPC(20:5) | [M+H]^+^ | **0.20** | 0.48 | 0.33 | 0.47 |
| 571.2903 | PI(16:0) | [M-H]^-^ | 0.48 | 0.59 | *0.71* | 0.65 |
| 575.1841 | Leucadenone A | [M+Cl]^-^ | **0.24** | 0.64 | 0.45 | 0.56 |
| 575.5035 | Mayolene-19 | [M+H]^+^ | 0.63 | *0.72* | 0.41 | *0.84* |
| 593.2741 | PI(18:3) | [M-H]^-^ | 0.43 | *0.79* | 0.45 | *0.81* |
| 595.2899 | PI(18:2) | [M-H]^-^ | 0.40 | *0.77* | 0.54 | *0.81* |
| 669.4520 | PA(34:3) | [M-H]^-^ | *0.80* | 0.48 | 0.69 | 0.63 |
| 671.4670 | PA(34:2) | [M-H]^-^ | 0.69 | 0.50 | *0.71* | 0.69 |
| 673.4828 | PA(34:1) | [M-H]^-^ | *0.71* | 0.49 | *0.82* | 0.58 |
| 697.4826 | PA(36:3) | [M-H]^-^ | 0.47 | 0.60 | *0.84* | 0.65 |
| 699.4986 | PA(36:2) | [M-H]^-^ | 0.62 | 0.56 | *0.83* | 0.59 |
| 711.4627 | OH-Chlorobactene glucoside | [M-H]^-^ | *0.71* | 0.43 | *0.77* | 0.63 |
| 712.5385 | GlcCer(d14:2/20:0(2OH)) | [M-H]^-^ | 0.45 | *0.74* | 0.67 | 0.62 |
| 714.5096 | PE(34:2) | [M-H]^-^ | 0.34 | 0.60 | *0.76* | 0.69 |
| 721.5049 | PG(32:0) | [M-H]^-^ | *0.87* | 0.37 | *0.77* | 0.63 |
| 736.4942 | PE(36:5) | [M-H]^-^ | **0.21** | 0.69 | 0.58 | 0.61 |
| 738.5101 | PE(36:4) | [M-H]^-^ | **0.27** | 0.63 | *0.70* | 0.62 |
| 740.5262 | PE(36:3) | [M-H]^-^ | **0.28** | 0.54 | 0.51 | 0.57 |
| 743.4887 | PG(34:3) | [M-H]^-^ | 0.53 | 0.43 | *0.76* | 0.59 |
| 745.5043 | PG(34:2) | [M-H]^-^ | 0.38 | 0.41 | *0.83* | 0.66 |
| 747.5203 | PG(34:1) | [M-H]^-^ | 0.68 | 0.37 | *0.82* | 0.57 |
| 751.4342 | PG(32:3) | [M+Cl]^-^ | 0.52 | 0.60 | *0.70* | 0.67 |
| 752.5071 | PI-Cer(d18:1/14:0) | [M+H]^+^ | *0.86* | 0.65 | 0.57 | 0.48 |
| 754.5375 | PC(34:4) | [M+H]^+^ | 0.35 | 0.37 | 0.41 | **0.16** |
| 756.5531 | PC(34:3) | [M+H]^+^ | **0.24** | 0.45 | 0.44 | 0.30 |
| 758.5688 | PC(34:2) | [M+H]^+^ | **0.24** | 0.49 | 0.50 | 0.43 |
| 776.5196 | PC(36:7) | [M+H]^+^ | **0.30** | 0.31 | 0.40 | **0.15** |
| 778.5354 | PC(36:6) | [M+H]^+^ | **0.21** | 0.49 | 0.45 | **0.16** |
| 780.5508 | PC(36:5) | [M+H]^+^ | **0.20** | 0.30 | 0.49 | **0.26** |
| 782.5677 | PC(36:4) | [M+H]^+^ | 0.38 | 0.47 | 0.46 | **0.30** |
| 800.5195 | PC(38:9) | [M+H]^+^ | **0.22** | 0.32 | 0.43 | **0.17** |
| 802.5350 | PC(38:8) | [M+H]^+^ | **0.21** | 0.42 | 0.39 | **0.21** |
| 808.5689 | PI-Cer(d20:1/16:0) | [M+H]^+^ | *0.74* | 0.48 | 0.48 | *0.78* |
| 818.5091 | (3'-sulfo)Galbeta-Cer(d18:1/16:0(2OH)) | [M+Na]^+^ | **0.30** | 0.43 | 0.38 | 0.33 |
| 831.5045 | PI(34:3) | [M-H]^-^ | *0.76* | 0.52 | 0.65 | 0.64 |
| 833.5217 | PI(34:2) | [M-H]^-^ | 0.65 | 0.56 | *0.74* | *0.74* |
| 835.5379 | PI(34:1) | [M-H]^-^ | 0.50 | 0.61 | *0.77* | 0.61 |
| 859.5373 | PI(36:3) | [M-H]^-^ | 0.47 | 0.60 | *0.77* | 0.57 |

* C: Clipper, G: Gairdner, H: Hindmarsh, M: Mundah.

Abbreviations: LPA: lysophosphatidic acid, LPC: lysophosphatidylcholine, LPE: lysophosphatidylethanolamine, PA: phosphatidic acid, PC: phosphatidylcholine, PE: phosphatidylethanolamine, PG: phosphatidylglycerol, PI: phosphatidylinositol, PI-Cer: phosphatidylinositol ceramide

**Table S4:** AUC values for discriminative lipid species found between root cap and zone of cell elongation (S2) root sections of control and salt-treated barley roots.

*AUC > 0.7* the peak was discriminative in control roots. **AUC < 0.3** the peak was discriminative in salt treated roots.

**Lipid tentative annotations were based on accurate precursor mass search (< 5 ppm) against the LIPID MAPS database (Fahy et al., 2007).*

| m/z | Name | Adduct | C | G | H | M |
| --- | --- | --- | --- | --- | --- | --- |
| 134.0473 | (+)-threo-2-Amino-3,4-dihydroxybutanoic acid | [M-H]^-^ | **0.11** | 0.50 | 0.63 | 0.5 |
| 145.0619 | 3,4-decadiene-6,8-diyn-1-ol | [M-H]^-^ | **0.25** | 0.53 | 0.49 | 0.69 |
| 179.0562 | Fuconic acid | [M-H]^-^ | 0.4 | 0.53 | **0.18** | 0.58 |
| 196.0384 | Aminoadipic acid | [M+Cl]^-^ | **0.3** | 0.49 | 0.38 | 0.5 |
| 235.1651 | 1,13-Dihydroxy-herbertene | [M+H]^+^ | 0.49 | **0.10** | 0.46 | *0.85* |
| 241.0128 | Lipoic acid | [M+Cl]^-^ | 0.54 | 0.45 | *0.78* | 0.55 |
| 243.0630 | Oxyresveratrol | [M-H]^-^ | 0.35 | 0.41 | *0.78* | 0.59 |
| 255.2334 | Palmitic acid | [M-H]^-^ | 0.54 | 0.45 | *0.81* | 0.47 |
| 275.0597 | Thysanone | [M-H]^-^ | **0.28** | 0.51 | 0.62 | 0.45 |
| 275.1257 | Dehydrofalcarinone | [M+Cl]^-^ | 0.55 | 0.49 | *0.83* | 0.54 |
| 277.1158 | (9R,13R)-1a,1b-dihomo-jasmonic acid | [M+K]^+^ | 0.46 | **0.18** | 0.50 | 0.61 |
| 277.2178 | alpha-Linolenic acid | [M-H]^-^ | 0.53 | 0.54 | *0.78* | 0.49 |
| 279.2335 | 10,13-Octadecadienoic acid | [M-H]^-^ | 0.51 | 0.54 | *0.81* | 0.51 |
| 281.2496 | 10-Octadecenoic acid | [M-H]^-^ | 0.41 | 0.50 | *0.81* | 0.47 |
| 283.0924 | Hemigossypol | [M+Na]^+^ | 0.67 | 0.50 | 0.35 | *0.81* |
| 299.0549 | 2'-Hydroxybiochanin A | [M-H]^-^ | 0.42 | 0.49 | *0.76* | 0.5 |
| 309.2809 | 10Z-Eicosenoic acid | [M-H]^-^ | 0.49 | 0.49 | *0.71* | 0.54 |
| 315.0689 | (+)-Bornyl-diphosphate | [M+H]^+^ | **0.03** | **0.06** | 0.48 | 0.5 |
| 323.0292 | Carthamidin | [M+Cl]^-^ | 0.36 | 0.48 | *0.76* | 0.53 |
| 331.1992 | 3a,17a-Dihydroxy-5b-androstane | [M+K]^+^ | 0.5 | 0.33 | **0.17** | 0.5 |
| 345.1382 | Dihydrokanakugiol | [M-H]^-^ | **0.29** | 0.57 | 0.62 | 0.42 |
| 357.1851 | 17-hydroxy-1-oxo-2,3-seco-androstan-3-oic acid | [M+Cl]^-^ | **0.22** | 0.51 | 0.70 | 0.45 |
| 359.2003 | 1-Acetoxy-2-hydroxy-16-heptadecyn-4-one | [M+Cl]^-^ | **0.22** | 0.51 | *0.71* | 0.44 |
| 361.2332 | 14R,21R-diHDHA | [M+H]^+^ | *0.75* | *0.71* | 0.47 | 0.58 |
| 389.2672 | 1alpha-hydroxy-25,26,27-trinorvitamin D3 24-carboxylic acid | [M+H]^+^ | 0.49 | 0.34 | 0.58 | *0.87* |
| 390.2751 | Anandamide (20:2, n-6) | [M+K]^+^ | 0.44 | 0.34 | 0.50 | *0.75* |
| 397.1707 | 7S,8S-epoxy-17R-HDHA | [M+K]^+^ | 0.37 | **0.13** | 0.32 | 0.49 |
| 398.2620 | Phytosphingosine 1-phosphate | [M+H]^+^ | 0.57 | *0.81* | 0.47 | 0.4 |
| 409.2373 | LPA(16:0) | [M-H]^-^ | 0.41 | 0.48 | *0.79* | 0.5 |
| 409.2563 | 6-deoxyerythronolide B | [M+Na]^+^ | 0.5 | *0.75* | 0.50 | 0.41 |
| 409.2665 | Dioctyl hexanedioate | [M+K]^+^ | 0.57 | *0.72* | 0.49 | 0.49 |
| 413.2106 | Docosatrienoate | [M-H]^-^ | 0.54 | 0.56 | *0.81* | 0.51 |
| 425.2831 | Sorbitan palmitate | [M+Na]^+^ | *0.73* | 0.31 | *0.83* | 0.67 |
| 433.1555 | Prebarbigerone | [M+Na]^+^ | 0.5 | **0.22** | 0.43 | 0.53 |
| 435.2527 | LPA(18:1) | [M-H]^-^ | 0.41 | 0.49 | *0.73* | 0.53 |
| 437.1360 | Antiarone K | [M+Cl]^-^ | 0.52 | 0.51 | *0.74* | 0.5 |
| 445.2873 | beta-tocotrienol | [M+Cl]^-^ | **0.19** | 0.50 | 0.60 | 0.46 |
| 452.2796 | LPE(16:0) | [M-H]^-^ | 0.56 | 0.49 | *0.73* | 0.55 |
| 459.1208 | 8-C-Methylvelloquercetin 3,5,3'-trimethyl ether | [M+Cl]^-^ | 0.55 | 0.53 | *0.72* | 0.52 |
| 461.1356 | (+)-Tephrorin A | [M+Cl]^-^ | 0.54 | 0.53 | *0.73* | 0.52 |
| 465.2633 | Minabeolide-2 | [M-H]^-^ | 0.44 | 0.50 | *0.71* | 0.49 |
| 483.2740 | PG(16:0) | [M-H]^-^ | 0.44 | 0.50 | *0.78* | 0.52 |
| 575.5035 | Mayolene-19 | [M+H]^+^ | 0.67 | *0.86* | 0.43 | 0.51 |
| 650.4386 | LPC(25:0(CHO)) | [M+H]^+^ | 0.49 | *0.72* | 0.36 | 0.48 |
| 669.4520 | PA(34:3) | [M-H]^-^ | 0.48 | 0.50 | *0.79* | 0.48 |
| 671.4670 | PA(34:2) | [M-H]^-^ | 0.41 | 0.46 | *0.78* | 0.5 |
| 673.4828 | PA(34:1) | [M-H]^-^ | 0.41 | 0.45 | *0.79* | 0.5 |
| 691.4356 | PA(36:6) | [M-H]^-^ | 0.55 | 0.52 | *0.82* | 0.55 |
| 693.4516 | PA(36:5) | [M-H]^-^ | 0.55 | 0.52 | *0.84* | 0.56 |
| 695.4681 | PA(36:4) | [M-H]^-^ | 0.41 | 0.51 | *0.80* | 0.56 |
| 697.4826 | PA(36:3) | [M-H]^-^ | 0.38 | 0.52 | *0.83* | 0.5 |
| 699.4986 | PA(36:2) | [M-H]^-^ | 0.47 | 0.50 | *0.80* | 0.5 |
| 712.4941 | PE(34:3) | [M-H]^-^ | 0.42 | 0.56 | *0.70* | 0.52 |
| 723.4996 | PA(38:4) | [M-H]^-^ | 0.53 | 0.50 | *0.75* | 0.52 |
| 743.4887 | PG(34:3) | [M-H]^-^ | 0.4 | 0.56 | *0.78* | 0.49 |
| 745.5043 | PG(34:2) | [M-H]^-^ | 0.34 | 0.52 | *0.77* | 0.51 |
| 747.5203 | PG(34:1) | [M-H]^-^ | 0.4 | 0.48 | *0.74* | 0.48 |
| 749.4174 | PG(32:4) | [M+Cl]^-^ | 0.39 | 0.47 | *0.72* | 0.52 |
| 752.5071 | PI-Cer(d18:1/14:0) | [M+H]^+^ | *0.91* | 0.68 | 0.51 | *0.79* |
| 754.5375 | PC(34:4) | [M+H]^+^ | 0.31 | *0.71* | 0.31 | 0.45 |
| 756.5531 | PC(34:3) | [M+H]^+^ | **0.17** | 0.54 | **0.27** | 0.52 |
| 758.5688 | PC(34:2) | [M+H]^+^ | **0.2** | 0.52 | 0.39 | 0.51 |
| 760.5847 | PC(34:1) | [M+H]^+^ | 0.43 | 0.55 | **0.29** | 0.64 |
| 773.4184 | PG(34:6) | [M+Cl]^-^ | 0.43 | 0.48 | *0.71* | 0.55 |
| 776.5196 | PC(36:7) | [M+H]^+^ | **0.3** | 0.64 | 0.40 | 0.38 |
| 778.5354 | PC(36:6) | [M+H]^+^ | **0.22** | 0.47 | 0.34 | 0.44 |
| 780.5508 | PC(36:5) | [M+H]^+^ | **0.25** | 0.38 | 0.46 | 0.47 |
| 798.5405 | PI-Cer(d18:0/16:0(2OH)) | [M+H]^+^ | 0.65 | *0.82* | **0.28** | 0.49 |
| 800.5195 | PC(38:9) | [M+H]^+^ | **0.13** | 0.55 | **0.30** | 0.41 |
| 802.5350 | PC(38:8) | [M+H]^+^ | **0.18** | 0.46 | 0.31 | 0.4 |
| 818.5091 | (3'-sulfo)Galbeta-Cer(d18:1/16:0(2OH)) | [M+Na]^+^ | 0.51 | 0.66 | **0.21** | 0.41 |
| 831.5045 | PI(34:3) | [M-H]^-^ | 0.52 | 0.48 | *0.78* | 0.54 |
| 833.5217 | PI(34:2) | [M-H]^-^ | 0.48 | 0.49 | *0.79* | 0.58 |
| 835.5379 | PI(34:1) | [M-H]^-^ | 0.39 | 0.50 | *0.74* | 0.5 |
| 859.5373 | PI(36:3) | [M-H]^-^ | 0.49 | 0.49 | *0.71* | 0.52 |

* C: Clipper, G: Gairdner, H: Hindmarsh, M: Mundah.

Abbreviations: LPA: lysophosphatidic acid, LPC: lysophosphatidylcholine, LPE: lysophosphatidylethanolamine, PA: phosphatidic acid, PC: phosphatidylcholine, PE: phosphatidylethanolamine, PG: phosphatidylglycerol, PI: phosphatidylinositol, PI-Cer: phosphatidylinositol ceramide

**Table S5:** AUC values for discriminative lipid species found between root cap and zone of cell maturation (S3) root sections of control and salt-treated barley roots.

*AUC > 0.75* the peak was discriminative in control roots. **AUC < 0.25** the peak was discriminative in salt treated roots.

**Lipid tentative annotations were based on accurate precursor mass search (< 5 ppm) against the LIPID MAPS database (Fahy et al., 2007).*

| ***m/z*** | **Name** | **Adduct** | **C** | **G** | **H** | **M** |
| --- | --- | --- | --- | --- | --- | --- |
| 134.0473 | (+)-threo-2-Amino-3,4-dihydroxybutanoic acid | [M-H]^-^ | **0.28** | 0.50 | 0.62 | 0.50 |
| 145.0619 | 3,4-decadiene-6,8-diyn-1-ol | [M-H]^-^ | 0.60 | 0.38 | *0.73* | *0.81* |
| 179.0562 | Fuconic acid | [M-H]^-^ | 0.65 | 0.32 | **0.28** | *0.72* |
| 225.0287 | Nemotinic acid | [M+Cl]^-^ | 0.48 | 0.58 | *0.74* | 0.56 |
| 235.1651 | 1,13-Dihydroxy-herbertene | [M+H]^+^ | 0.50 | **0.22** | 0.44 | *0.84* |
| 241.0128 | Lipoic acid | [M+Cl]^-^ | 0.46 | 0.54 | *0.83* | 0.49 |
| 243.0630 | Oxyresveratrol | [M-H]^-^ | **0.10** | 0.36 | *0.75* | 0.56 |
| 255.2334 | Palmitic acid | [M-H]^-^ | 0.52 | 0.66 | *0.84* | 0.38 |
| 265.1781 | all-trans-7-hydroxyhexadeca-2,4,8,10-tetraenoic acid | [M+H]^+^ | 0.53 | *0.73* | 0.50 | **0.24** |
| 273.1804 | Beta-estradiol | [M+H]^+^ | 0.51 | *0.73* | 0.53 | **0.29** |
| 275.1257 | Dehydrofalcarinone | [M+Cl]^-^ | 0.53 | 0.49 | *0.78* | 0.48 |
| 277.1158 | (9R,13R)-1a,1b-dihomo-jasmonic acid | [M+K]^+^ | 0.47 | **0.20** | 0.49 | *0.72* |
| 277.2178 | alpha-Linolenic acid | [M-H]^-^ | 0.52 | *0.77* | *0.82* | 0.43 |
| 279.2335 | 10,13-Octadecadienoic acid | [M-H]^-^ | 0.55 | *0.76* | *0.82* | 0.44 |
| 281.2496 | 10-Octadecenoic acid | [M-H]^-^ | 0.52 | *0.75* | *0.80* | 0.41 |
| 283.0924 | Hemigossypol | [M+Na]^+^ | *0.85* | 0.66 | **0.11** | *0.91* |
| 295.2285 | 10-keto-12Z-octadecenoic acid | [M-H]^-^ | *0.79* | 0.65 | 0.55 | 0.51 |
| 299.0549 | 2'-Hydroxybiochanin A | [M-H]^-^ | 0.43 | 0.64 | *0.77* | 0.45 |
| 301.0334 | 6-Hydroxykaempferol | [M-H]^-^ | 0.52 | *0.72* | 0.66 | 0.45 |
| 309.2809 | 10Z-Eicosenoic acid | [M-H]^-^ | 0.55 | 0.61 | *0.73* | 0.50 |
| 315.0689 | (+)-Bornyl-diphosphate | [M+H]^+^ | **0.05** | **0.13** | 0.46 | 0.52 |
| 323.0292 | Carthamidin | [M+Cl]^-^ | 0.37 | 0.59 | *0.73* | 0.50 |
| 325.1262 | Diisopentyl thiomalate | [M+Cl]^-^ | *0.85* | 0.63 | 0.57 | *0.81* |
| 331.1109 | 2'-Hydroxymatteucinol | [M+H]^+^ | *0.73* | **0.26** | **0.17** | *0.81* |
| 331.1992 | 3a,17a-Dihydroxy-5b-androstane | [M+K]^+^ | 0.50 | 0.38 | **0.11** | 0.64 |
| 357.1851 | 17-hydroxy-1-oxo-2,3-seco-androstan-3-oic acid | [M+Cl]^-^ | 0.33 | 0.61 | *0.70* | 0.50 |
| 361.2096 | 12R-HETrE | [M+K]^+^ | 0.51 | 0.52 | **0.20** | 0.69 |
| 389.2672 | 1alpha-hydroxy-25,26,27-trinorvitamin D3 24-carboxylic acid | [M+H]^+^ | 0.50 | *0.74* | 0.58 | *0.98* |
| 390.2751 | Anandamide (20:2, n-6) | [M+K]^+^ | 0.53 | *0.78* | 0.51 | *0.92* |
| 397.1707 | 7S,8S-epoxy-17R-HDHA | [M+K]^+^ | 0.42 | **0.17** | **0.12** | 0.67 |
| 398.2620 | Phytosphingosine 1-phosphate | [M+H]^+^ | 0.60 | *0.92* | 0.52 | **0.30** |
| 399.1443 | 2'-Hydroxypiscerythrinetin | [M+H]^+^ | 0.61 | 0.43 | **0.19** | 0.65 |
| 401.2690 | Ophiobolin A | [M+H]^+^ | 0.50 | *0.74* | 0.50 | 0.49 |
| 401.2788 | 24:3(15Z,18Z,21Z) | [M+K]^+^ | 0.50 | *0.76* | 0.50 | 0.50 |
| 409.2373 | LPA(16:0) | [M-H]^-^ | 0.41 | 0.68 | *0.84* | 0.46 |
| 409.2563 | 6-deoxyerythronolide B | [M+Na]^+^ | 0.51 | *0.81* | 0.50 | 0.31 |
| 409.2665 | Dioctyl hexanedioate | [M+K]^+^ | 0.50 | *0.80* | 0.53 | 0.52 |
| 413.2106 | Docosatrienoate | [M-H]^-^ | 0.45 | *0.75* | *0.87* | 0.45 |
| 425.2831 | Sorbitan palmitate | [M+Na]^+^ | 0.52 | **0.21** | 0.47 | *0.83* |
| 433.1555 | Prebarbigerone | [M+Na]^+^ | 0.49 | **0.28** | 0.39 | 0.52 |
| 433.2370 | LPA(18:2) | [M-H]^-^ | 0.42 | 0.63 | *0.78* | 0.50 |
| 435.2527 | LPA(18:1) | [M-H]^-^ | 0.52 | 0.61 | *0.71* | 0.50 |
| 437.1360 | Antiarone K | [M+Cl]^-^ | 0.54 | 0.62 | *0.73* | 0.48 |
| 445.2873 | beta-tocotrienol | [M+Cl]^-^ | **0.30** | 0.54 | 0.69 | 0.50 |
| 447.1872 | 1-palmitoylglycerone 3-phosphate | [M+K]^+^ | 0.42 | 0.35 | **0.17** | 0.50 |
| 459.1208 | 8-C-Methylvelloquercetin 3,5,3'-trimethyl ether | [M+Cl]^-^ | 0.53 | 0.63 | *0.77* | 0.49 |
| 461.1356 | (+)-Tephrorin A | [M+Cl]^-^ | 0.53 | 0.64 | *0.75* | 0.48 |
| 465.2633 | Minabeolide-2 | [M-H]^-^ | 0.55 | 0.65 | *0.72* | 0.45 |
| 483.2740 | PG(16:0) | [M-H]^-^ | 0.46 | 0.67 | *0.74* | 0.47 |
| 571.2903 | PI(16:0) | [M-H]^-^ | 0.43 | 0.69 | *0.78* | 0.48 |
| 575.1841 | Leucadenone A | [M+Cl]^-^ | **0.14** | 0.53 | 0.43 | 0.47 |
| 575.5035 | Mayolene-19 | [M+H]^+^ | 0.65 | *0.90* | 0.62 | 0.41 |
| 650.4386 | LPC(25:0(CHO)) | [M+H]^+^ | *0.74* | *0.87* | 0.41 | 0.44 |
| 669.4520 | PA(34:3) | [M-H]^-^ | 0.37 | 0.64 | *0.86* | 0.44 |
| 671.4670 | PA(34:2) | [M-H]^-^ | 0.38 | 0.62 | *0.85* | 0.42 |
| 673.4828 | PA(34:1) | [M-H]^-^ | 0.45 | 0.55 | *0.76* | 0.48 |
| 691.4356 | PA(36:6) | [M-H]^-^ | 0.42 | 0.64 | *0.88* | 0.50 |
| 693.4516 | PA(36:5) | [M-H]^-^ | 0.43 | 0.68 | *0.88* | 0.47 |
| 695.4681 | PA(36:4) | [M-H]^-^ | 0.43 | 0.65 | *0.84* | 0.47 |
| 697.4826 | PA(36:3) | [M-H]^-^ | 0.49 | *0.71* | *0.84* | 0.43 |
| 699.4986 | PA(36:2) | [M-H]^-^ | 0.59 | 0.59 | *0.78* | 0.49 |
| 712.4941 | PE(34:3) | [M-H]^-^ | 0.56 | *0.71* | *0.75* | 0.48 |
| 712.5385 | GlcCer(d14:2/20:0(2OH)) | [M-H]^-^ | 0.48 | 0.67 | *0.80* | 0.53 |
| 714.5096 | PE(34:2) | [M-H]^-^ | 0.59 | *0.72* | *0.72* | 0.50 |
| 723.4996 | PA(38:4) | [M-H]^-^ | 0.61 | 0.58 | *0.75* | 0.50 |
| 736.4942 | PE(36:5) | [M-H]^-^ | 0.51 | *0.73* | *0.73* | 0.49 |
| 738.5101 | PE(36:4) | [M-H]^-^ | 0.55 | *0.72* | 0.68 | 0.51 |
| 743.4887 | PG(34:3) | [M-H]^-^ | 0.48 | *0.80* | *0.79* | 0.43 |
| 745.5043 | PG(34:2) | [M-H]^-^ | 0.55 | *0.74* | *0.75* | 0.43 |
| 752.5071 | PI-Cer(d18:1/14:0) | [M+H]^+^ | *0.88* | 0.56 | 0.38 | *0.74* |
| 756.5531 | PC(34:3) | [M+H]^+^ | 0.37 | 0.51 | **0.22** | 0.36 |
| 758.5688 | PC(34:2) | [M+H]^+^ | 0.43 | 0.50 | **0.28** | 0.40 |
| 780.5508 | PC(36:5) | [M+H]^+^ | 0.47 | *0.74* | 0.36 | 0.40 |
| 782.5463 | PI-Cer(d18:0/16:0) | [M+H]^+^ | 0.60 | *0.78* | 0.50 | 0.49 |
| 798.5405 | PI-Cer(d18:0/16:0(2OH)) | [M+H]^+^ | 0.68 | *0.85* | 0.34 | 0.38 |
| 800.5195 | PC(38:9) | [M+H]^+^ | 0.34 | 0.65 | 0.42 | **0.24** |
| 802.5350 | PC(38:8) | [M+H]^+^ | 0.43 | 0.54 | 0.33 | **0.27** |
| 804.5492 | PC(38:7) | [M+H]^+^ | 0.54 | 0.52 | 0.40 | **0.29** |
| 812.6160 | PC(38:3) | [M+H]^+^ | 0.51 | *0.71* | 0.51 | 0.36 |
| 818.5091 | (3'-sulfo)Galbeta-Cer(d18:1/16:0(2OH)) | [M+Na]^+^ | 0.54 | 0.65 | **0.25** | **0.30** |
| 831.5045 | PI(34:3) | [M-H]^-^ | 0.40 | 0.68 | *0.87* | 0.50 |
| 833.5217 | PI(34:2) | [M-H]^-^ | 0.45 | *0.70* | *0.85* | 0.51 |
| 835.5379 | PI(34:1) | [M-H]^-^ | 0.49 | *0.73* | *0.80* | 0.48 |
| 857.5215 | PI(36:4) | [M-H]^-^ | 0.54 | 0.65 | *0.78* | 0.50 |
| 859.5373 | PI(36:3) | [M-H]^-^ | 0.56 | 0.66 | *0.74* | 0.47 |

* C: Clipper, G: Gairdner, H: Hindmarsh, M: Mundah.

Abbreviations: LPA: lysophosphatidic acid, LPC: lysophosphatidylcholine, LPE: lysophosphatidylethanolamine, PA: phosphatidic acid, PC: phosphatidylcholine, PE: phosphatidylethanolamine, PG: phosphatidylglycerol, PI: phosphatidylinositol, PI-Cer: phosphatidylinositol ceramide

**Table S6:** Description of candidate genes of interest (GOI) chosen from the Glycerophospholipid Metabolism KEGG Pathway (https://www.genome.jp/kegg/pathway/map/map00564.html).

| **Gene code** | **KEGG Ec number** | **Transcript ID** | **Chromosome location** | | | **Description** | |
| --- | --- | --- | --- | --- | --- | --- | --- |
| GOI 1 | 3.1.1.5 | HORVU2Hr1G122470.1 | chr2H: | Start:  Stop: | 753,282,710  753,289,542 | | K06130 - lysophospholipase II (LYPLA2) |
| GOI 2 | 3.1.1.5 | HORVU3Hr1G023960.1 | chr3H | Start:  Stop: | 90,192,353  90,196,331 | | PTHR10655//PTHR10655:SF34 - Lysophospholipase-related // Subfamily not named |
| GOI 3 | 3.1.4.4 | HORVU5Hr1G084740.1 | chr5H | Start:  Stop: | 573,176,086  573,183,958 | | PTHR18896:SF60 - Phospholipase D Delta |
| GOI 4 | 3.1.4.46 | HORVU3Hr1G079900.5 | chr3H | Start:  Stop: | 585,699,789  585,703,782 | | PTHR22958//PTHR22958:SF9 - Related to multifunctional Cyclin-dependent kinase-related // subfamily not named |
| GOI 5 | 4.1.1.65 | HORVU4Hr1G088470.27 | chr4H | Start:  Stop: | 640,534,471  640,538,737 | | Phosphatidylserine decarboxylase / PS decarboxylase |

**Table S7:** Selected candidate target and reference genes, primers, and amplicon characteristics.

GOI: gene of interest, Tm: melting temperature, bp: base pairs, *E*: amplification efficiency, *R^2^*: coefficient of determination.

| **Gene** | **Gene code** | **Amplicon sequence  (5’ – 3’)** | **Amplicon length (bp)** | **Product Tm (°C)** | ***E*** | ***R^2^*** |
| --- | --- | --- | --- | --- | --- | --- |
| GAPDH | GAPDH | CCCTTCATCACCACCGAGTA | 102 | 82.5 | 105.6 | 0.995 |
|  |  | CAGCGTCTTGTCGTCTTTGA |  |  |  |  |
| SALM | SALM | GGGAGATTGGCTCTGGAAAT | 110 | 79.5 | 102.6 | 0.998 |
|  |  | GCCTCTTGGGTGTGGTTTAG |  |  |  |  |
| HORVU2Hr1G122470 | GOI 1 | TGTGTGTGGGTTCAGTCAGG | 118 | 85.0 | 103.3 | 0.998 |
|  |  | AATGACCGACGAACCGAAAG |  |  |  |  |
| HORVU3Hr1G023960 | GOI 2 | GGGCACTATACCGTTCCAG | 171 | 84.0 | 104.8 | 0.999 |
|  |  | CACTCAATTTCGGCACAACC |  |  |  |  |
| HORVU4Hr1G088470 | GOI 3 | ATCCGCAGTCTCAAACAAGG | 121 | 84.5 | 105.0 | 0.994 |
|  |  | GCTCAATCATGGTTCCTGGAC |  |  |  |  |
| HORVU3Hr1G079900 | GOI 4 | TACATGCAGCACCTCATGGG | 131 | 87.5 | 100.8 | 0.997 |
|  |  | ACCACTTGGCTGCTCAGATC |  |  |  |  |
| HORVU5Hr1G084740 | GOI 5 | AACGGTCAGGCATTACTACC | 111 | 81.5 | 104.6 | 0.994 |
|  |  | TCCTCGCAATCAAGTCAACATC |  |  |  |  |

**Table S8:** The elemental composition of roots of four barley cultivars grown under control and salt (150 mM NaCl) conditions.

| **Cultivar** | **Clipper** | | | | **Gairdner** | | | | **Hindmarsh** | | | | **Mundah** | | | |
| --- | --- | --- | --- | --- | --- | --- | --- | --- | --- | --- | --- | --- | --- | --- | --- | --- |
| **Element** | C | | S | | C | | S | | C | | S | | C | | S | |
| K [× 10^3^] | 34.8 ± | 3.9 | **25.3 ±** | **0.6** | 49.5 ± | 4.5 | **28.7 ±** | **2.4** | 25.2 ± | 0.8 | **20.8 ±** | **2.2** | 28.2 ± | 2.2 | **17.6 ±** | **1.0** |
| Na [× 10^3^] | 1.7 ± | 0.2 | **21 ±** | **0.8** | 2.7 ± | 0.3 | **19 ±** | **1.1** | 2.9 ± | 0.4 | **19.3 ±** | **1.1** | 2.9 ± | 0.4 | **19.8 ±** | **2.1** |
| P [× 10^3^] | 6.4 ± | 0.7 | 7.5 ± | 0.1 | 9.7 ± | 1.0 | 8 ± | 0.5 | 6.1 ± | 0.2 | 6.5 ± | 0.3 | 5.1 ± | 0.7 | 6.7 ± | 0.7 |
| Mg [× 10^2^] | 13.9 ± | 2.6 | 18 ± | 0.6 | 16 ± | 1.7 | 13.6 ± | 0.8 | 9.7 ± | 0.3 | 10.9 ± | 0.7 | 9.6 ± | 1.6 | 11.2 ± | 1.3 |
| Ca [× 10^2^] | 9.2 ± | 0.9 | 8.6 ± | 0.2 | 9.4 ± | 0.9 | 6.4 ± | 0.3 | 12.6 ± | 2.2 | 10.6 ± | 0.8 | 11.5 ± | 1.0 | 9.1 ± | 1.1 |
| Fe [× 10] | 10.4 ± | 1.7 | **7.2 ±** | **0.7** | 15.9 ± | 0.7 | **12.4 ±** | **0.9** | 9.6 ± | 1.7 | 10.6 ± | 1.5 | 14.8 ± | 0.6 | 12 ± | 1.2 |
| Zn | 54.9 ± | 6.2 | 58.7 ± | 1.5 | 62.1 ± | 6.6 | 47.9 ± | 2.6 | 64.8 ± | 10.1 | 56.5 ± | 3.4 | 44.8 ± | 7.2 | 47.6 ± | 5.9 |
| Mn | 28 ± | 3.9 | 39.4 ± | 1.4 | 30.6 ± | 2.5 | 31.8 ± | 2.2 | 26.3 ± | 1.9 | 36.5 ± | 5.9 | 23.9 ± | 2.7 | 33.1 ± | 3.5 |
| Cu | 4.5 ± | 0.4 | 4.7 ± | 0.1 | 4.5 ± | 0.6 | 4.2 ± | 0.4 | 5.1 ± | 0.4 | 5.4 ± | 0.2 | 4.7 ± | 0.6 | 5 ± | 0.6 |

Data points represent mean ± SEM, n = 3. The content in mg kg^-1^ DW of 9 elements was measured from root tissue of 48-old seedlings using ICP-MS. The data were transformed to better represent data. Values that were found to be significantly different with a *P* < 0.05 are indicated in bold. DW: dry weight.

**Fahy E, Sud M, Cotter D, Subramaniam S. 2007.** LIPID MAPS online tools for lipid research. *Nucleic Acids Res* **35**(Web Server issue): W606-612.
