# Supplementary figures and images for: Comparative spatial lipidomics analysis reveals cellular lipid remodelling in different developmental zones of barley roots in response to salinity
Source: Plant Cell Environ. 2019 Nov 29;43(2):327–43. doi: 10.1111/pce.13653 (PMC7063987; doi:10.1111/pce.13653)

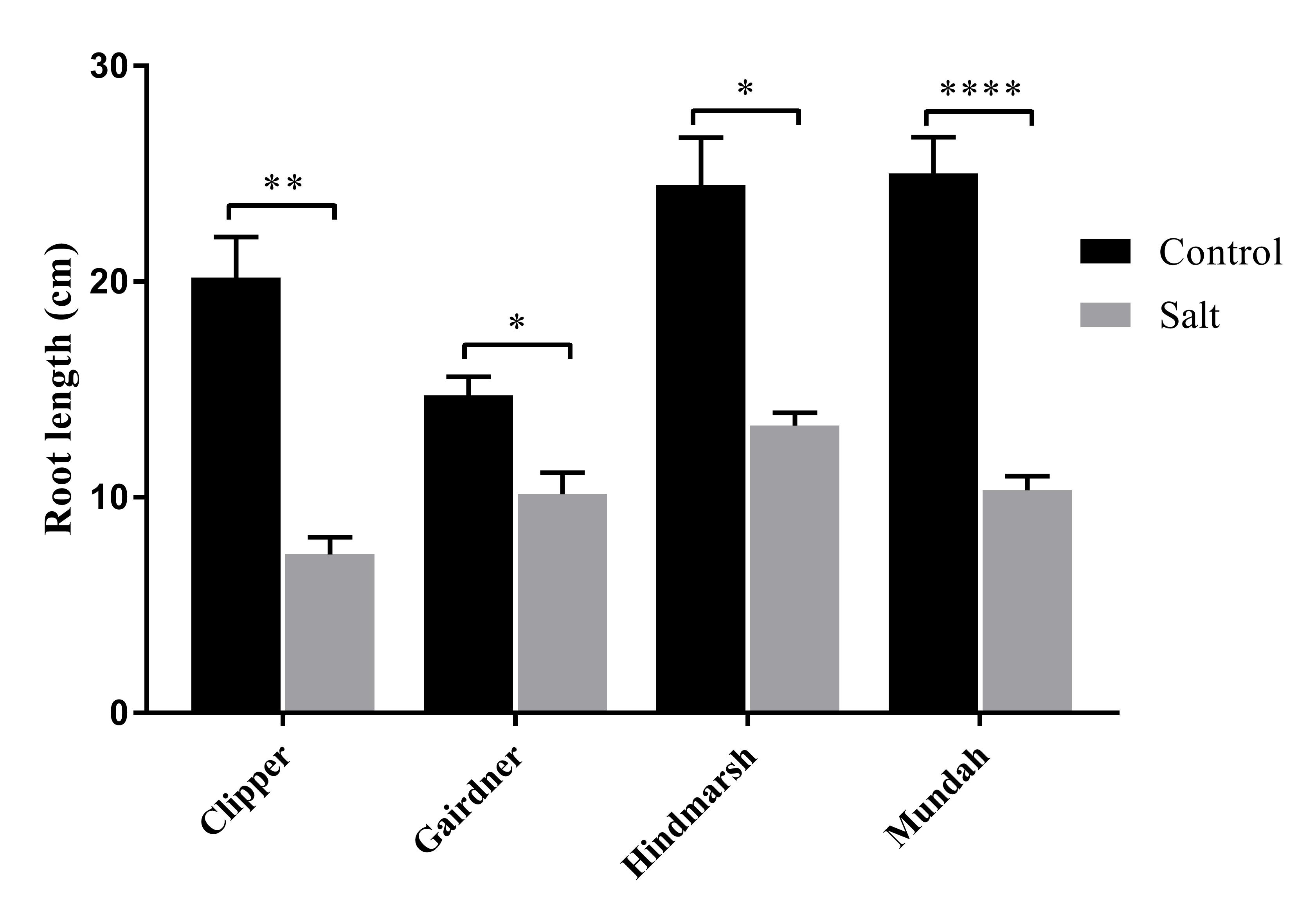

Supplement: Supplementary file 5 — Figure S1. Supporting information [file PCE-43-327-s005.jpg]

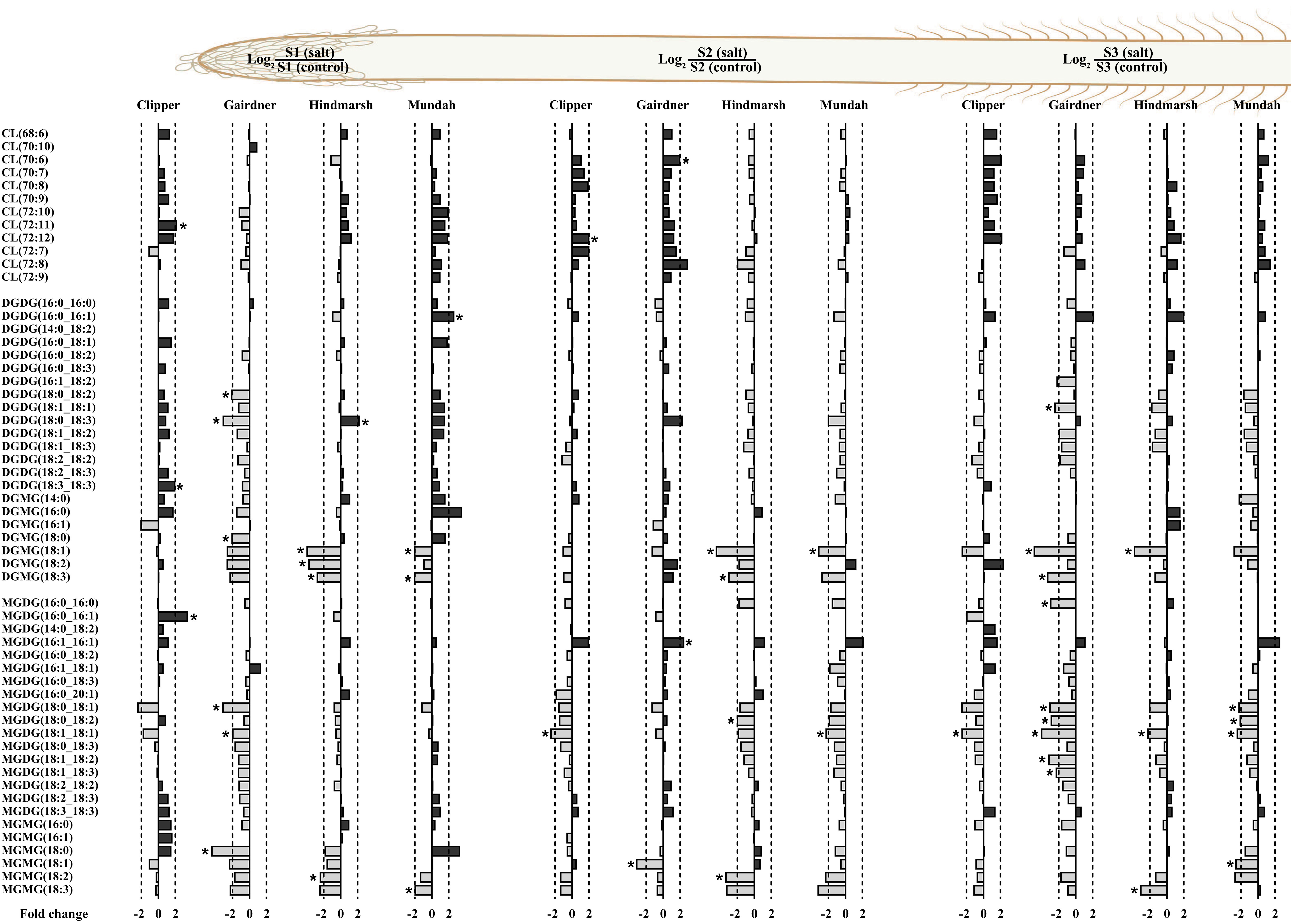

Supplement: Supplementary file 6 — Figure S2. Supporting information [file PCE-43-327-s006.jpg]

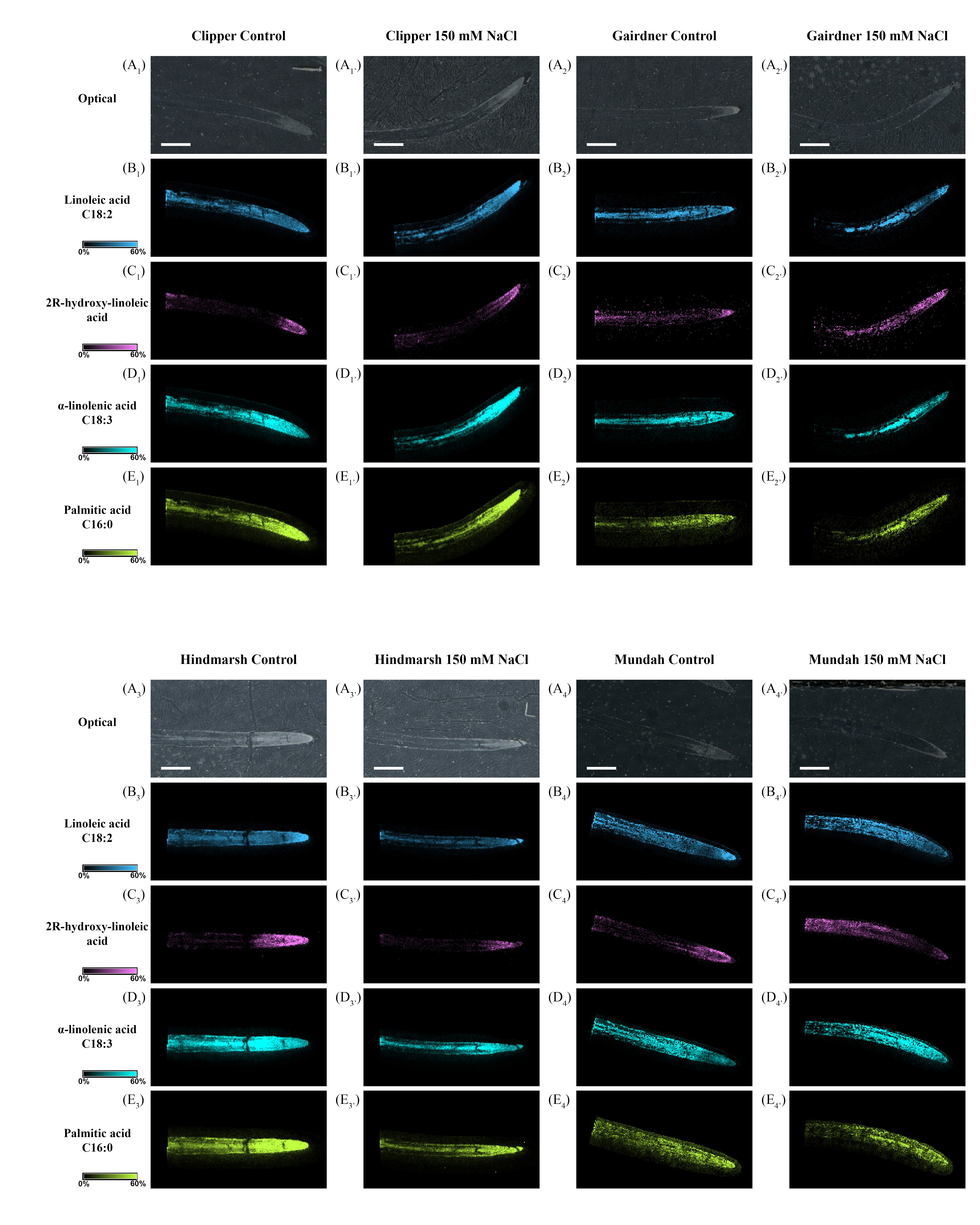

Supplement: Supplementary file 7 — Figure S3. Supporting information [file PCE-43-327-s007.jpg]
